# Primary EBV Infection Induces an Expression Profile Distinct from Other Viruses but Similar to Hemophagocytic Syndromes

**DOI:** 10.1371/journal.pone.0085422

**Published:** 2014-01-17

**Authors:** Samantha K. Dunmire, Oludare A. Odumade, Jean L. Porter, Juan Reyes-Genere, David O. Schmeling, Hatice Bilgic, Danhua Fan, Emily C. Baechler, Henry H. Balfour, Kristin A. Hogquist

**Affiliations:** 1 Center for Immunology, University of Minnesota Medical School, Minneapolis, Minnesota, United States of America; 2 Department of Laboratory Medicine and Pathology, University of Minnesota Medical School, Minneapolis, Minnesota, United States of America; 3 Department of Medicine, University of Minnesota Medical School, Minneapolis, Minnesota, United States of America; 4 Biostatistics and Bioinformatics Core, University of Minnesota Medical School, Minneapolis, Minnesota, United States of America; 5 Department of Pediatrics, University of Minnesota Medical School, Minneapolis, Minnesota, United States of America; The University of North Carolina at Chapel Hill, United States of America

## Abstract

Epstein-Barr Virus (EBV) causes infectious mononucleosis and establishes lifelong infection associated with cancer and autoimmune disease. To better understand immunity to EBV, we performed a prospective study of natural infection in healthy humans. Transcriptome analysis defined a striking and reproducible expression profile during acute infection but no lasting gene changes were apparent during latent infection. Comparing the EBV response profile to multiple other acute viral infections, including influenza A (influenza), respiratory syncytial virus (RSV), human rhinovirus (HRV), attenuated yellow fever virus (YFV), and Dengue fever virus (DENV), revealed similarity only to DENV. The signature shared by EBV and DENV was also present in patients with hemophagocytic syndromes, suggesting these two viruses cause uncontrolled inflammatory responses. Interestingly, while EBV induced a strong type I interferon response, a subset of interferon induced genes, including *MX1, HERC5*, and *OAS1*, were not upregulated, suggesting a mechanism by which viral antagonism of immunity results in a profound inflammatory response. These data provide an important first description of the response to a natural herpesvirus infection in humans.

## Introduction

Epstein-Barr virus (EBV) is a herpesvirus that causes lifelong infection in humans. EBV is estimated to be a causative agent in 1% of all human cancers, [1] including various lymphomas, and nasopharyngeal and gastric carcinomas; [2] and to contribute to autoimmune diseases such as systemic lupus erythematosus (SLE) [3] and multiple sclerosis. [4] Nonetheless, the most common consequence of EBV infection is infectious mononucleosis (IM). [5] Primary acquisition of the virus by children before puberty does not generally cause recognizable symptoms, but those who become infected during or after adolescence have a high likelihood of developing IM. EBV infection is trending older, particularly in developed countries, [6] and IM represents a growing health issue. There is currently no vaccine and no effective treatment for EBV infection. IM is distinct from acute infection with other viruses in that it is characterized by a lengthy incubation period and severe lymphocytosis. The incubation period for EBV is about six weeks in length, [7] contrasting starkly with symptomatic viral illnesses such as influenza where the incubation period is only a few days. In addition, the lymphocytosis observed in IM reflects a profound activation and expansion of EBV specific, and to a lesser extent, bystander CD8+ T cells [8], [9].

In order to gain insight into EBV pathogenesis, we examined the host response to EBV using a genomic approach. Systems biology is emerging as an important tool for understanding the human response to infection that can provide novel means of diagnosis and insight into disease mechanisms. [10] Recent studies have reported genomic changes occurring in B cells infected with EBV *in vitro* [11] or EBV+ Burkitt Lymphoma cell lines. [12] However, there is a dearth of understanding in current research about the host gene changes that occur during natural viral infection with EBV in otherwise healthy humans and whether or not these infections lead to long lasting changes in the immune system. We sought to address these issues by performing transcriptomic studies on human subjects participating in a prospective study of primary EBV infection acquired naturally. We then compared these data with gene expression analyses of other acute viral infections, systemic illnesses, and interferon (IFN) driven immune responses. We found the human immune response to EBV has a distinct and reproducible gene expression signature that more closely resembles the dysregulated innate immune responses observed during cytokine storm illness than what is seen during other acute viral infections. Furthermore, we identified a subset of genes that represent likely targets of viral immune evasion mechanisms.

## Materials and Methods

### Ethics statement

All participants gave written informed consent and the University of Minnesota Institutional Review Board approved all protocols used.

### Design of prospective study

We recruited 546 healthy undergraduate volunteers from the University of Minnesota residence halls in 2006 and 2007 as recently described. [13] EBV naïve subjects were identified as those lacking IgG antibodies against EBV viral capsid antigen (EBV VCA IgG) and EBV nuclear antigen 1 (EBNA1). Of the 202 eligible EBV-naïve subjects, 143 (71%) were enrolled in the prospective study. For enrolled participants, blood and oral washings were collected at least every 8 weeks during the academic year in addition to an electronic monitoring journal to track development of symptoms between visits. Subjects with symptoms consistent with acute primary EBV infection were asked to come to the clinical virology research clinic for both a physical exam and laboratory-confirmation of primary EBV infection via Monospot, EBV serology, and viral titer in the oral cavity or blood. 66 subjects experienced primary infection (46%), of which only 6 were asymptomatic. [13] Primary EBV infection was defined as a positive EBV antibody test and the presence of EBV DNA in the blood and/or oral compartment of a subject who was previously negative for both EBV antibodies and EBV DNA. All participants continued pre-scheduled follow-up visits after seroconversion.

### Samples collection and handling

Peripheral blood samples were obtained from subjects via venipuncture and collected in 10 ml purple-top EDTA Vacutainer® tubes (Fisher Scientific). 200μl of blood was used for DNA extraction and HLA typing. Blood Peripheral blood mononuclear cells (PBMCs) were isolated by Accuspin™ System-Histopaque®-1077 (Sigma-Aldrich) density gradient centrifugation per manufacturer’s instructions. PBMC counts were recorded post-ficoll purification of whole blood during each collection and stored as detailed below. Once pelleted, cells were frozen in 1×10^7^ cells/ml aliquots in a cryopreservative solution containing 90% FBS and 10% dimethysulfoxide (DMSO) (Sigma-Aldrich). Samples were allowed to slowly freeze at −80°C overnight and then transferred to liquid nitrogen for storage until needed. Cells were rapidly thawed in a 37°C water bath, diluted to 10 ml in RPNK media supplemented with 50U/ml benzonase (Novagen) (RPNK media: RPMI 1640 (Cellgro) supplemented with 10% FBS (Atlanta Biologicals), 2% Penicillin – Streptomycin (5000U/ml, 5000 μg/ml respectively, GIBCO, Invitrogen) and 1% L-glutamine (29.2 mg/ml, GIBCO)). Cells were then counted using a hemocytometer and divided into separate fractions for flow cytometry or RNA processing.

### RNA extraction

For each sample, 1–2×10^6^ PMBCs were used for each RNA extraction. Cells were first homogenized using QIAshredder columns (Qiagen) per the manufacturer’s instructions. RNA was then extracted using RNeasy kit (Qiagen) with on column DNase step (Qiagen) per the manufacturer’s instructions. RNA was then quantified using a Nanodrop 2000/2000c spectrophotometer (Thermo Scientific) and kept frozen at −80°C.

### Microarray

200ng of RNA per sample was amplified for microarray analysis using the Illumina TotalPrep RNA Amplification Kit (Ambion) per the manufacturer’s instructions. Tagged transcripts were then sent to the microarray facility of the BioMedical Genomics Center at the University of Minnesota for hybridization to an Illumina Sentrix BeadChip HumanRef-8 v3. Preinfection and latent samples were hybridized to Illumina HumanHT-12 v4 Expression Beadchips.

### Microarray analysis and heatmap generation

Raw data from [14], [15], [16], [17], [18], [19], [20] were obtained from the GEO database and analyzed independently as described. Signal values from all microarrays were imported into the microarray analysis program GeneSpring (Agilent). Signal values were normalized by percentile and filtered to eliminate noise from the data. Fold changes were calculated as the difference between acute infection samples and baseline (in the event that the subjects had baselines) or healthy controls (for clinical studies with no available baselines). Studies with before and after samples were subjected to a paired *t*-test with Bonferonni multiple tests correction. Studies which used healthy controls were subjected to an unpaired unequal variance *t*-test (Welch) with Bonferonni multiple tests correction. Log_2_ transformed fold changes were exported from GeneSpring into a *.txt file. These values were imported into the open source program Cluster 3.0, [21] which clustered the genes hierarchically using a Pearson non-averaged correlation and average linkage. Heatmaps were then visualized using the program Java Treeview [22].

### Ingenuity Pathway Analysis

Pathway analysis was performed using Ingenuity Pathway Analysis (IPA) software (Ingenuity System). Gene sets were screened to limit the number of genes utilized for analysis. For the acute gene set, the 464 significantly changed genes previously described were used. 53 genes with potential long-term changes were used from the latent analysis. Log_2_ transformed fold changes for all relevant genes were imported into the software using their Illumina probe identifiers and were then associated with their appropriate gene symbol by IPA. The software then used these genes and their fold changes to determine which gene sets had statistically significant importance in each of the cases. Canonical pathways were assigned based on the number of genes present in our geneset for each pathway, the level at which these genes were up or down regulated, and the likelihood these trends would occur by chance by using the multiple test correction Fisher’s exact test. Results are reported in this work as the Benjamini-Hochberg p-value or ratio.

### Gene Set Enrichment Analysis

Gene Set Enrichment Analysis (GSEA) was performed on whole chip acute and latent infection microarray data using GSEA software (Broad Institute). Analysis was performed per Broad Institute instructions.

### Fluorescence activated cell sorting

Frozen PBMCs from baseline and acute IM timepoints from 4 subjects were thawed and stained to be sorted into four different subsets: CD8 T cells (CD3+, CD56−, CD8+), NK cells (CD56+, CD3−), B cells (CD19+, CD20+), and monocytes (CD14/CD16). Subsets were sorted into 1.5 ml tubes pre-coated with media using a FACS Aria under BSL-2 sorting conditions. Cells were washed with PBS and RNA was extracted as described above.

### cDNA synthesis and PCR analysis using SuperArray

100ng of RNA was used with the SuperScript III Platinum Two-Step qRT-PCR Kit (Invitrogen) to generate cDNA. Samples were then stored at −20°C. 43 genes were selected for analysis by PCR from a larger list of changed genes in IM subjects and other acute viral infections that comprised relevant functional groupings as assessed by IPA. 384-well SuperArray plates pre-coated with primers for the desired 43 genes plus 5 controls were obtained as a custom order from SABiosciences. 10 μL of cDNA per subject was used with RT2 Real-time SYBR green/Rox PCR master mix (SABiosciences) for qRT-PCR analysis. Products were detected using an ABI Prism 7900HT Sequence Detection System (Applied Biosystems). The genes ACTB, B2M, and RPL13A were used as housekeeping genes during the calculation of fold changes as they had previously been shown not to change significantly during acute EBV infection (data not shown). Fold changes were calculated as: 2∧ (Δ Acute Housekeeping Control – Baseline Housekeeping Control)/2∧(Δ Acute Gene of Interest – Baseline Gene of Interest).

### Blinded study and K-means clustering

Subject samples for the blinded gene signature study were chosen by H.H.B Jr and RNA extraction was performed by O.A.O to ensure S.K.D was blinded to sample identity. cDNA synthesis, qRT-PCR and analysis were performed by S.K.D. Sample cycle thresholds were imported into Cluster 3.0 as a text file and a K-means clustering was performed on the samples with 3 groupings and 10,000 iterations to provide a sufficient number of successful solutions from the algorithm.

### Statistical analysis

Except for where Genespring (Agilent) or IPA (Ingenuity Systems) was used, most other statistical analysis was performed using Prism software (Graphpad). MedCalc (MedCalc Software) was used for comparison of Pearson correlation coefficients. Comparisons between groups were performed with either an unpaired two-tailed *t*-test or a one-way ANOVA with a p-value less that 0.05 as the cutoff for statistical significance. Pearson correlation coefficients were generated with Prism by comparing pairs of relevant groupings.

### Accession numbers

Gene expression data is available from the Gene Expression Omnibus. The data are part of superseries GSE45924 composed of two subseries, GSE45918 and GSE45919.

## Results

### Prospective analysis of EBV infection in young adults

EBV naïve subjects were recruited from freshman classes at the University of Minnesota during 2006 and 2007. Enrolled study subjects were screened for exposure to EBV on an ongoing basis by testing blood or throat wash samples taken every four to eight weeks. Samples were evaluated for EBV genomes by qPCR and/or antibodies to viral antigens. Of 143 subjects followed in the study, 66 experienced primary EBV infection during their undergraduate years [13].

Due to the long incubation period, the exact date of viral acquisition is not known. Instead, date of symptom onset was used for timing, since this date was well defined for each subject. For acute microarray analysis, we chose eight subjects with peripheral blood samples taken near the onset of symptoms, and compared them to samples taken at least three months before infection. These eight subjects plus an additional two were chosen for analysis of latency, with latent samples being defined as at least >200 days after infection (Table S1). Baseline and latency samples were chosen from timepoints when the subjects did not report symptoms of any other illness and had not reported illness for at least two weeks prior. Further analysis was performed by PCR on an additional 44 subjects following initial evaluation of these eight by microarray as described below.

### Primary EBV infection produces a distinct gene expression signature in peripheral blood during acute infection but not during latency

**Figure 1:**
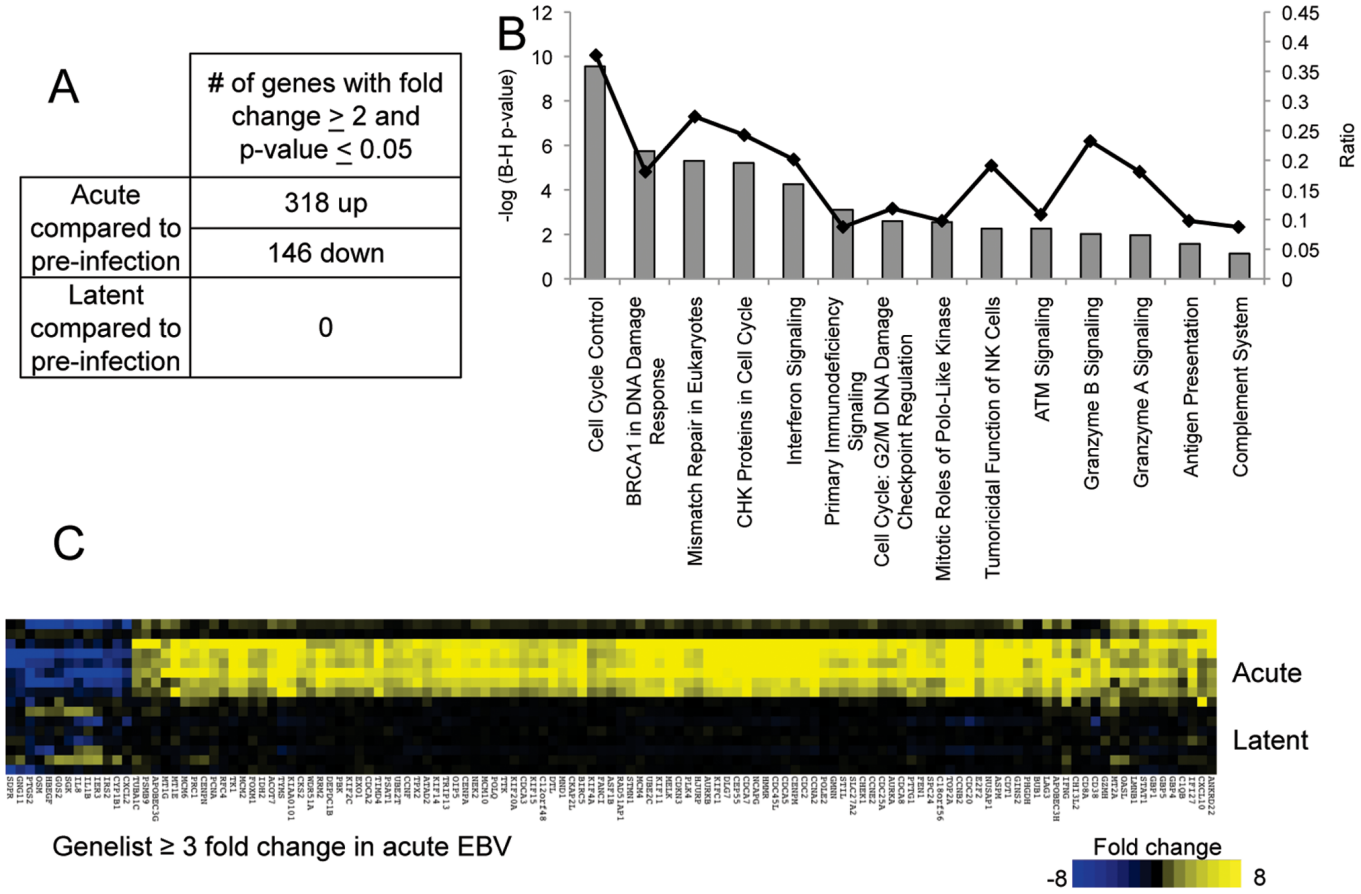
A distinct gene expression profile is apparent during acute EBV infection, but not latent infection. (A) Microarray analysis was performed on pre-infection, acute, and latent timepoints for the 10 subjects with primary EBV infection (listed in Table S1). 464 genes were shown to be significantly changed during the primary response to EBV at a fold change of ≥2 and a p-value ≤0.05. No genes were significantly changed during the latent phase of infection using the same criteria. (B) Ingenuity Pathway Analysis of the 464 acute genes revealed 14 pathways that were enriched amongst the genes that changed during primary EBV. These had a significant p-value (the negative log is shown) following evaluation with the Benjamini-Hochberg multiple tests correction. (C) A heatmap representation of the highest (≥3 fold) gene changes during the acute and latent stages of EBV infection.

Microarray analysis was performed on peripheral blood mononuclear cells (PBMCs) from subjects described above. Each was compared with his or her own healthy pre-infection sample. Gene expression fold changes greater than 2, with a p-value of ≤0.05 with Bonferonni multiple tests correction were considered significant. For subjects presenting with acute IM, this analysis yielded a list of 464 genes that changed more than two fold (Figure 1A and Table S2). 318 genes were upregulated and 146 genes were downregulated. Of the genes changed during acute IM, one of the largest constituent functional groups was found to be cell cycle by Ingenuity Pathway Analysis (Figure 1B). Likewise, pathways such as mismatch repair, DNA damage response, and ATM signaling, are associated with highly proliferative populations. Genes associated with interferon signaling were significantly enriched in the acute gene list, reflecting the host response to viral infection. Finally, pathways representing various immune functions, such as granzyme signaling, antigen presentation, NK function, and complement were enriched in the acute EBV gene list (Figure 1B).

Studies in mice showed that latent herpesvirus infection protected hosts from subsequent bacterial infection, suggesting that persistent infection with herpesviruses may have a beneficial role in their human hosts[23], [24]. Thus, we wanted to determine if long-term gene changes were measureable in human subjects after they had acquired primary EBV. In contrast to acute infection, subjects with latent infections yielded no significantly changed genes compared with pre-infection (Figures 1A/C, and data not shown). This approach is highly sensitive as each subject’s latent infection sample was compared with his or her own pre-infection sample. Nonetheless, there were no gene changes that met significance criteria, even *IFNG*, which was shown to be elevated in mice latently infected with a murine gammaherpesvirus [23].

### Different cell types contribute to the gene expression signature during IM

**Figure 2:**
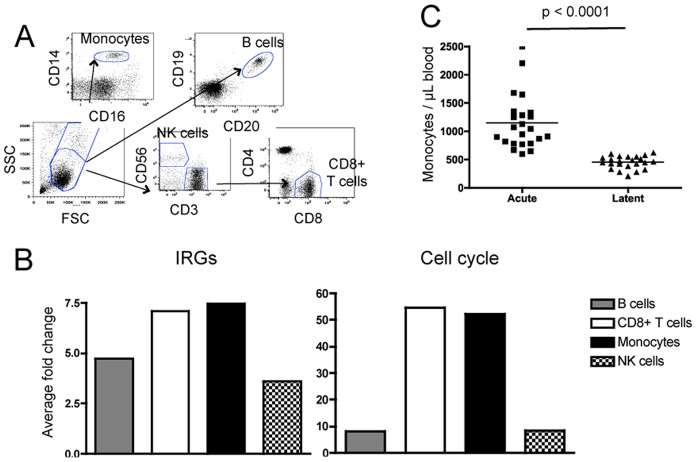
CD8+ T cells and monocytes show upregulation of key gene groups during acute infection. Quantitative PCR analysis was performed on RNA prepared from sorted cells from peripheral blood of four subjects before and during acute EBV infection. (A) This panel shows the staining and sorting strategy for separation of CD8 T cells, B cells, NK cells, and monocytes. (B) Each subject’s respective cell type was compared with his or her own cell type at the preinfection time point. All acute samples were taken from subjects within a week of onset of symptoms. The average fold change of both type I and type II IRG as well as cell cycle genes for four subjects is shown. A paired t-test was used for statistical analysis. (C) Monocyte numbers were determined from complete blood counts of subjects at the acute and latent phases of EBV infection.

We chose to perform analysis of whole PBMCs in this study to facilitate the comparison to data published on other infections and disorders (see below). However, we still sought to understand which cellular components were representing the distinct aspects of the signature observed in acute EBV infection. Thus, we measured gene expression in sorted CD8+ T cells, natural killer (NK) cells, monocytes, and B cells (Figure 2A) from 4 subjects using a quantitative PCR array of 43 genes selected from the acute microarray analysis (Figure S1). These genes comprised the three functional groups: Type I interferon regulated genes (IRGs), type II IRGs, and cell cycle/metabolism genes.

All cell types displayed ≥3 fold upregulation of type I IRGs, suggesting that the interferon response during primary EBV infection is systemic, not limited to a particular cell type (Figure 2B). The cell cycle aspect of the gene expression signature, however, was highest in monocytes and CD8+ T cells. We found this result surprising given the fact that an increase in the number of monocytes had not been previously appreciated in IM literature. Complete blood counts from our subjects indicate that monocyte numbers were indeed elevated during IM (Figure 2C). Recent evidence has emerged showing IFNγ can induce monopoeisis, [25] thus, our data are consistent with a role for IFNγ in the proliferation of monocytes during primary EBV infection.

Human peripheral blood has been shown to have different subgroups of monocytes, which may be broadly described as inflammatory M1 monocytes or immunoregulatory M2 monocytes. [26] Given the fact that M2 monocytes are induced by IL-10 and EBV produces a viral IL-10 homolog we explored the possibility that the increase in monocytes numbers is accompanied by a shift in the M1 to M2 ratio during IM. However, no major change in expression of CD163 or HLA-DR (commonly used to distinguish M1 and M2) was found on monocytes during acute infection (data not shown). Thus, although monocytes are increased during acute infection, they may represent a heterogeneous mix of cells that does not fit the established M1/M2 paradigm.

### Comparison of EBV to other viral infections and SLE

**Figure 3:**
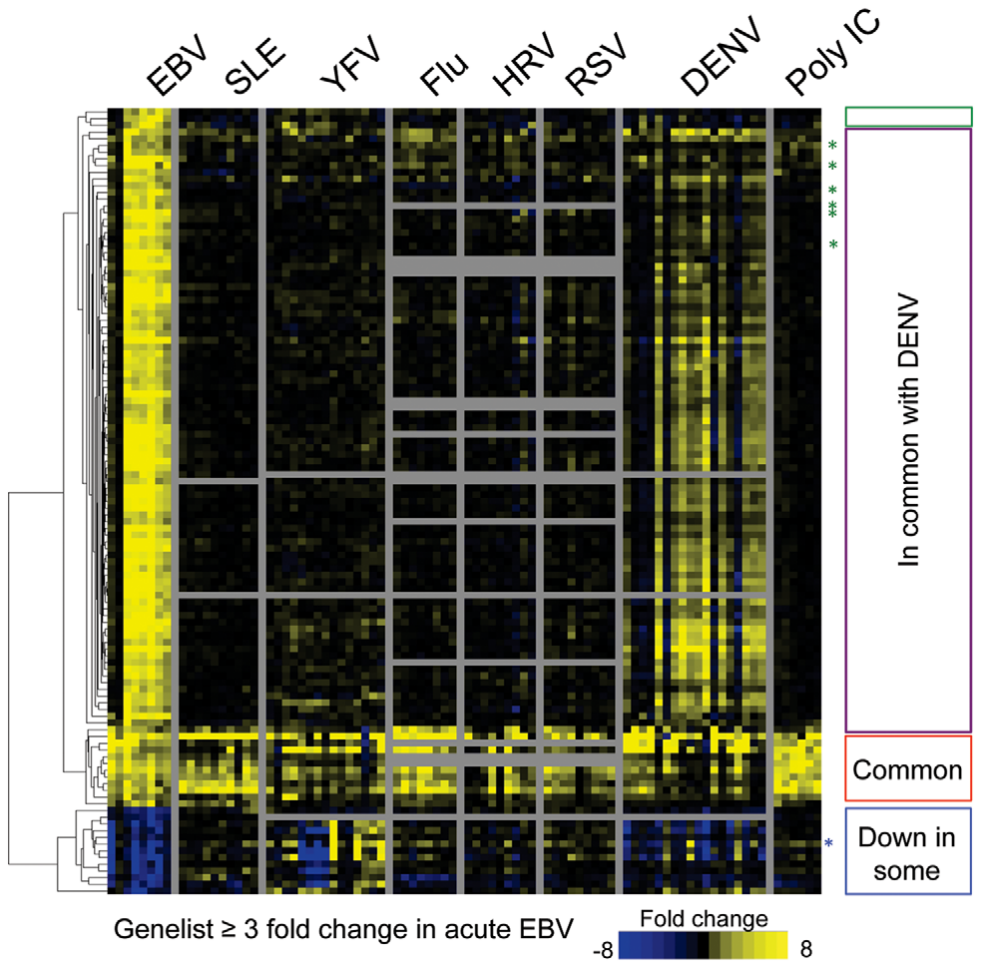
Primary Epstein-Barr virus infection causes distinct expression patterns in comparison to other interferon driven responses. Heatmap shows a list of the genes up/down regulated 3-fold during acute infection with EBV in comparison to subjects with other viral infections (YFV, Flu, HRV, RSV, and DENV), subjects injected with Poly IC, or patients with SLE. The color intensity represents fold changes in gene expression in comparison to either each subjects’ own healthy baseline (EBV, Flu, HRV, RSV, and Poly IC) or in comparison to healthy controls (SLE and DENV). Data is arranged as a hierarchical clustering of genes. The genes shown were derived from analysis of EBV subjects (fold change ≥3 and met a statistical significance cutoff of p-value of ≤0.05 with Bonferonni multiple-tests correction, please see Table S2 for fold change ≥2). Each column represents a single subject. Horizontal gray bars indicate that the gene of interest was not present in the analyzed dataset used for comparison. Vertical gray bars separate disease groupings. Colored boxes on the right represent specific clusters of genes discussed in the text: green box and green asterisks – EBV unique, purple box – genes in common between EBV and DENV, red – genes upregulated in all situations, blue – genes down-regulated in EBV and some other situations.

We compared our data on EBV with published data sets from five other acute viral infections including influenza A virus (Flu), human rhinovirus (HRV), respiratory syncitial virus (RSV), [14] dengue fever virus (DENV), [15] and yellow fever virus vaccine strain (YFV). [16] Additional comparison was performed against samples from patients with the autoimmune disease SLE, because EBV had been suggested to be an etiologic agent of SLE[27] and SLE patients have been found to express IRGs. [17] Data were also compared with subjects who were administered polyinosinic:polycytidylic acid (Poly IC), as an inducer of a classic type I interferon response. [18] A heatmap of the EBV gene set with a fold change (FC) ≥3 provided a good visual representation of this comparison (Figure 3), although a more complete gene list (FC ≥2) can be found in Table S2. Young adults presenting with acute EBV infection have a distinct gene expression pattern from those with other acute viral infections, SLE, or subjects administered Poly IC (Figure 3). Four main points are apparent from the heatmap:

#### IRGs are induced in all viral infections

The greatest similarity between EBV and other acute viral illnesses was found amongst the genes that are outlined in the red box in Figure 3 (also listed in Table S2). These genes are composed almost exclusively of IRGs. IRGs were defined using the online database Interferome and by comparison to PBMCs stimulated by IFN *in vitro* (data not shown). Not surprisingly, such genes were also upregulated by Poly IC, and were a dominant element of the SLE signature (Figure 3). [17] Interestingly, only a minority, 21% of the EBV gene signature was comprised of IRGs. This is markedly lower than Flu and YFV, where 48% and 65%, respectively, of the gene changes were IRGs (Figure S2). Further analysis via classification of type I IRGs and type II IRGs, showed there was fairly equal representation between the two IFN types for EBV. Flu and YFV, however, were dominated by type I IRGs, with more than 80% being regulated by type I IFN (Figure S2). Thus, while primary EBV exhibited marked upregulation of IRGs, they did not represent a dominant aspect of the signature, and reflected an enrichment of a type II IFN response in addition to the more typical type I IFN response.

#### EBV unique genes

The heatmap in Figure 3 also shows a set of genes increased in primary EBV infection but not other infections (Figure 3, green box and green asterisks). We defined a total of 59 genes that were “EBV unique” in comparison to all other acute viral infections examined using a 2-fold increase cutoff for EBV (Table S4). Given this finding, we wondered if primary EBV infection could be distinguished from other acute common viral illnesses. We conducted a small, blinded analysis of clinical samples. Analysis of 43 genes correctly distinguished acute EBV illness from non-EBV viral illnesses (Figure S3).

#### Response to EBV shares similarity to Dengue

From this comparison, we also observed that EBV shares little overall similarity to other acute infections with the exception of DENV. Almost 60% of the genes upregulated ≥3 by EBV, and nearly all of the downregulated genes were also similarly up/down regulated in a majority of DENV patients (Figure 3, purple and blue boxes). A possible pitfall to comparing disparate human infections and conditions is that sample timing may be different in infections with different incubation times. Nonetheless, the large group of “in common with DENV” genes was not upregulated at any time point even up to 28 days following YFV or Poly IC (Figure S4). Thus, upregulation of these genes seems peculiar to EBV and DENV.

#### Genes down-regulated after EBV

The heatmap in Figure 3 also identifies a discrete set of genes downregulated in acute EBV infection (Figure 3, blue box). Many of these genes are inflammatory or stress response genes expressed by monocytes, including *IL1B, IL8, MIP2A*, and *OSM* (Table S3). The down-regulation of *IL1B* and *IL8* was confirmed by real-time PCR in 70 timepoints from 52 subjects (data not shown). This downregulation is particularly surprising for *IL1B*, which is considered a type I IRG and therefore one might expect it would be upregulated during viral infection. Indeed, *IL1B* was generally upregulated during antiviral responses, including subjects with Flu, HRV, and RSV, and subjects treated with Poly IC or SLE patients (blue asterisk in Figure 3).

### EBV and DENV resemble inflammatory syndromes

**Figure 4:**
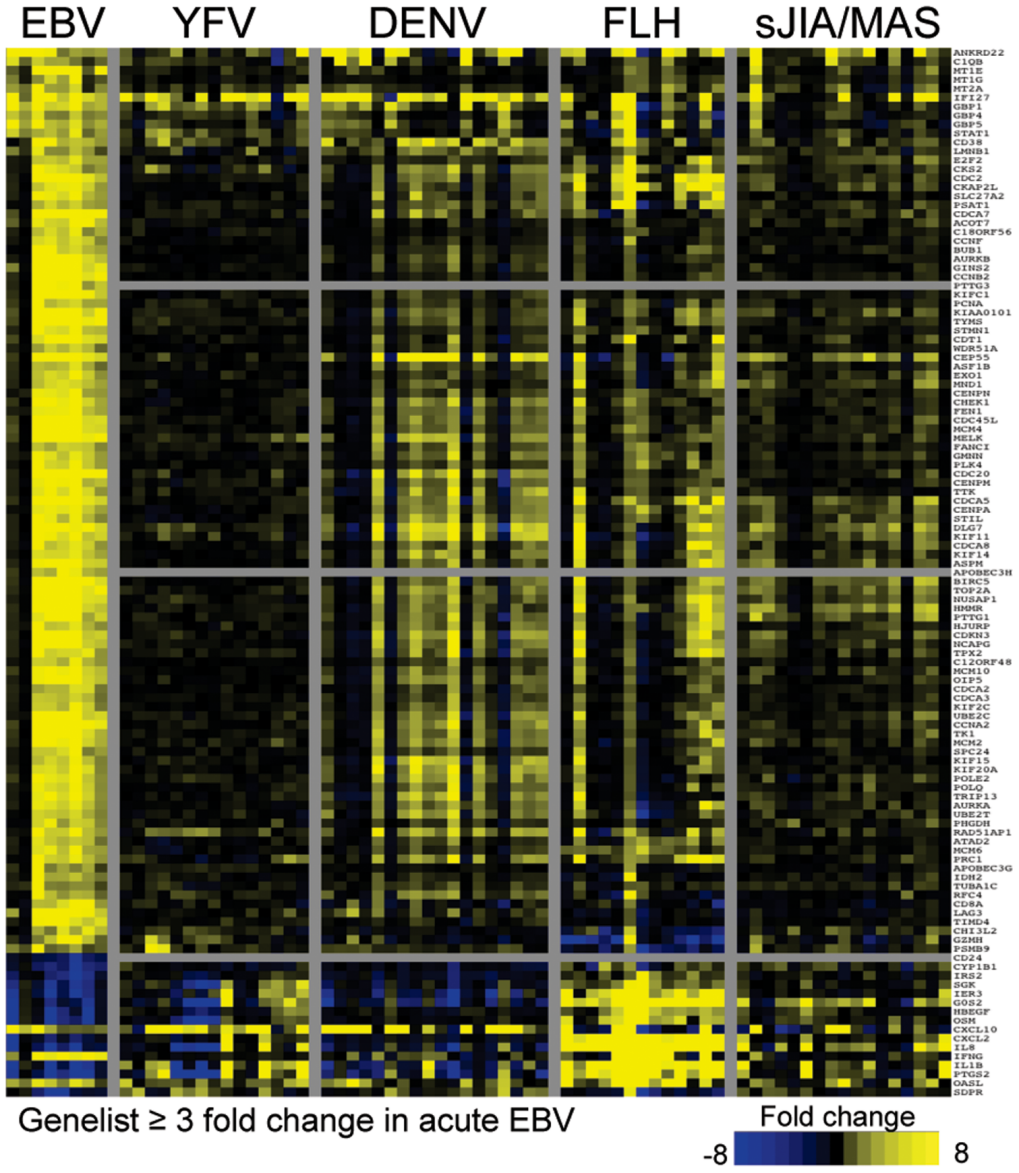
EBV has high similarity to DENV and inflammatory syndromes. This heatmap compares the genes that change during primary EBV with DENV, and two syndromes: juvenile idiopathic arthritis with macrophage inflammatory syndrome (sJIA/MAS) and familial lymphocytic histiocytosis (FLH). The fold changes in gene expression are in comparison to either each subjects’ own healthy baseline (EBV) or healthy controls (DENV, sJIA/MAS and FLH). Genes shown had a fold change ≥3 in acute EBV and met a statistical significance cutoff of p-value of ≤0.05 with Bonferonni multiple-tests correction.

Due to the surprising difference between the EBV/DENV signature and that observed in other acute viral infections, we theorized that perhaps the signature would more closely resemble that observed during inflammatory syndromes. To this end we examined microarray data obtained from subjects with familial hemophagocytic lymphohistiocytosis (FLH) [19] or subjects presenting with systemic onset juvenile arthritis (sJIA) who were later confirmed to have subclinical macrophage activation syndrome (MAS). [20] Both of these syndromes may be classified under the larger umbrella of hemophagocytic diseases, but are distinct in their origins. FLH is most commonly caused by mutations associated with degranulation genes, especially perforin (*PRF1*) and the syntaxins. The genetic basis of MAS is less well understood but the vast majority of patients first present with sJIA. [28] Indeed, many of the gene changes shared between EBV and DENV were also found in patients with these inflammatory disorders (Figure 4). This suggests that the host defense to EBV and DENV more closely resembles uncontrolled inflammation that it does during other antiviral response. This would help explain the lack of similarity between the EBV response and what is observed in other acute viral infections or an IFN associate autoimmune disease.

### Type I and type II IFN response kinetics differ

**Figure 5:**
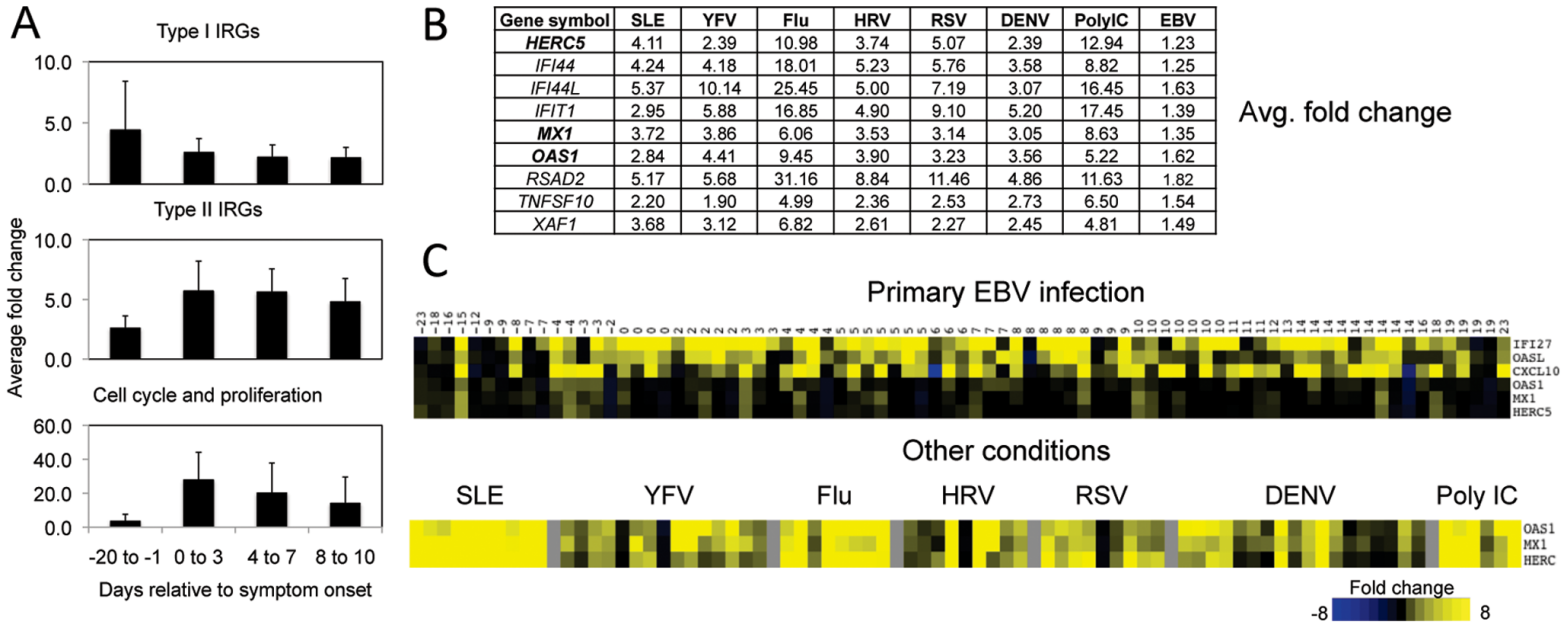
Type I IRGs are slightly enriched before onset of symptoms, but some IRGs show no change. (A) Superarray analysis by qPCR was performed on 70 timepoints from 52 subjects to assess the time course of gene expression. Average fold changes for three groups of genes (Type I IRG, Type II IRG, and Cell Cycle as defined in Figure S1) were determined for samples collected within the indicated time blocks. Fold changes are compared with healthy baselines. Error bars show the standard deviation. All three gene groupings had a p-value<0.05 when evaluated by ANOVA. (−20 days to −1 days N = 11; 0 days to 3 days N = 12; 4 days to 7 days N = 17; 8 days to 10 days N = 16). (B) The table shows the average fold change for nine select type I IRGs derived from microarray analysis for SLE, YFV, Flu, HRV, RSV, DENV, Poly IC, and EBV. (C) Heatmaps showing fold change as determined by qPCR for *OAS1*, *MX1*, and *HERC5* at multiple timepoints in EBV infection (top panel) or in other situations (bottom panel).

Although we cannot know precisely when subjects were infected with EBV, we were able to “stage” the subjects relative to the date of symptom onset. In the heatmaps shown, the subjects are ordered according to their symptom onset date. It is apparent that set B genes (IRGs) were upregulated in two of the pre-symptomatic subjects, but the other major aspect of the signature was absent (Figure 4). To determine if this reflected temporally distinct aspects of the signature, we extended our analysis to multiple timepoints from a larger number of subjects. We employed the PCR array of 43 genes described above, representing genes in three main functional groups: Type I IRGs, type II IRGs, and cell cycle/metabolism. With this assay, we extended our analysis to include data from 70 different timepoints between −23 days and 20 days relative to symptom onset, comprising samples from 50 subjects. The trend from these analyses suggests that on average, type I IRG changes occur earlier during infection (including during the incubation period) than either type II IRG or cell cycle genes (Figure 5A).

### EBV fails to induce key interferon response genes

While a type I interferon response clearly occurs during EBV infection, we were surprised to observe that *MX1*, which is a classic IFN induced GTP-binding protein, [29] was not upregulated in any of the primary EBV subjects. Upon further examination, we defined nine IRGs that were upregulated in all other viral infections, with Poly IC, and in SLE patients; but not in the EBV subjects (Figure 5B). To determine if we may have missed an early spike in gene expression as suggested from the analysis above, we selected three of these *MX1*, *OAS1*, and *HERC5* and performed real-time PCR on 70 timepoints from 50 subjects. As expected, these genes were upregulated in every other acute infection data set examined and in SLE (Figure 5C bottom rows). However, none were upregulated more than two fold in any of the pre-symptom or post-symptom timepoints we examined (Figure 5C). Selective antagonism of specific IRGs or interferon signaling is common in viral infections. [30], [31] However, one would typically expect this to only be observed in virally infected cells (in our case, relatively rare circulating B cells). Instead the selective antagonism of IRGs we observed is occurring in the context of a systemic interferon response. Thus, we favor the hypothesis that EBV produces a soluble molecule that selectively antagonizes IRG gene expression in uninfected cells.

### The IRG signature of SLE is not similar to EBV

**Figure 6:**
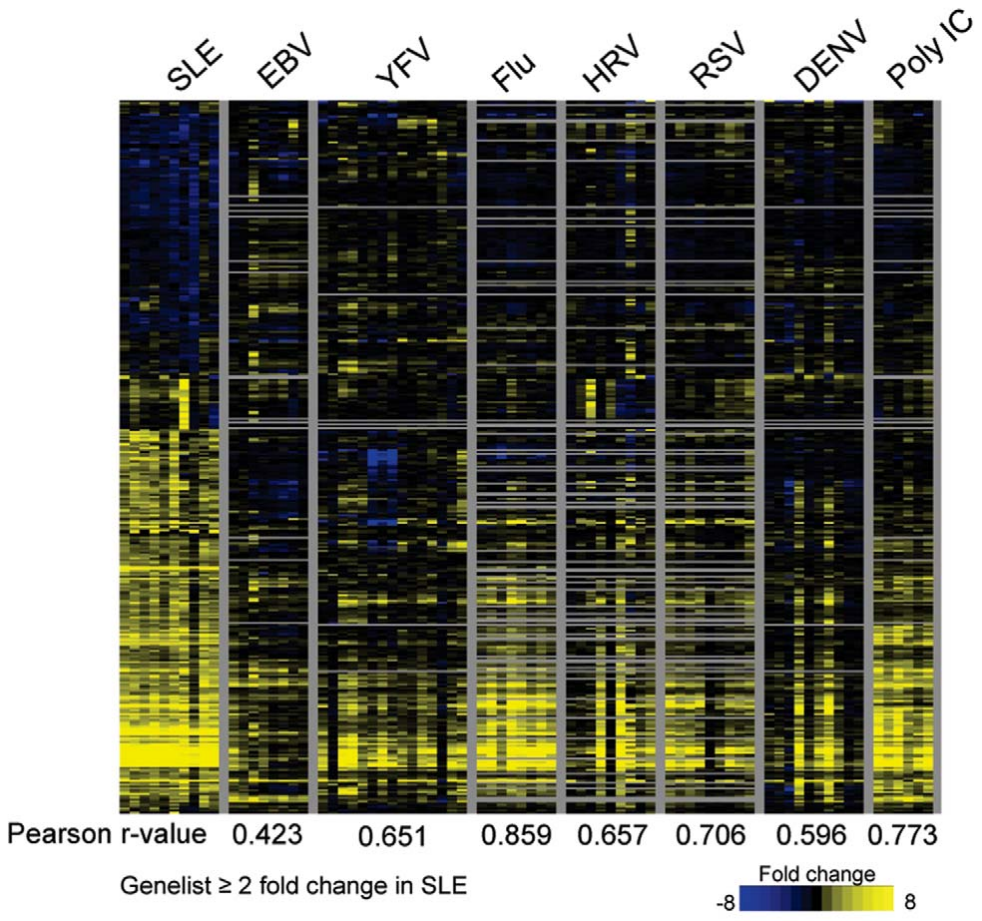
SLE has high similarity to influenza and Poly IC but not EBV or DENV. The heatmap lists the genes that are up/down regulated ≥2 fold in SLE patients compared with subjects with viral infections or who were injected with Poly IC. The fold changes in gene expression are in comparison to healthy controls (SLE and DENV) or to each subjects’ own healthy baseline (EBV, YFV, Flu, HRV, and RSV). Pearson r-values indicating degree of similarity between SLE and each of the other conditons are shown at the bottom of the heatmap.

EBV has been suggested to be involved in the pathogenesis of SLE. [32] A significantly higher proportion of SLE patients are infected with EBV than the general population [33], [34] and they have higher viral loads and antibody titers. [35], [36] Some SLE patients also have high IFN signatures. [17] Thus, it was hypothesized that the interferon response may be driven by EBV [32] and EBV derived nucleic acids could fuel type I IFN production via plasmacytoid dendritic cells. [37] We sought to investigate this link at the gene expression level. Patients with high IFN signatures [17] were compared with healthy controls using the same statistical criteria described for EBV above. Interestingly, we found that SLE had the least similarity to EBV as demonstrated by the low Pearson r-value (Figure 6). Conversely, both Flu and Poly IC had high levels of correlation. In particular the nine IRGs that are selectively antagonized during EBV infection are strongly upregulated in SLE patients (Figure 5B). Thus, our data do not support the notion that EBV reactivation contributes to the interferon gene signature in SLE.

### Type II IRG expression correlates with CD8 T cell expansion in primary EBV expression

**Figure 7:**
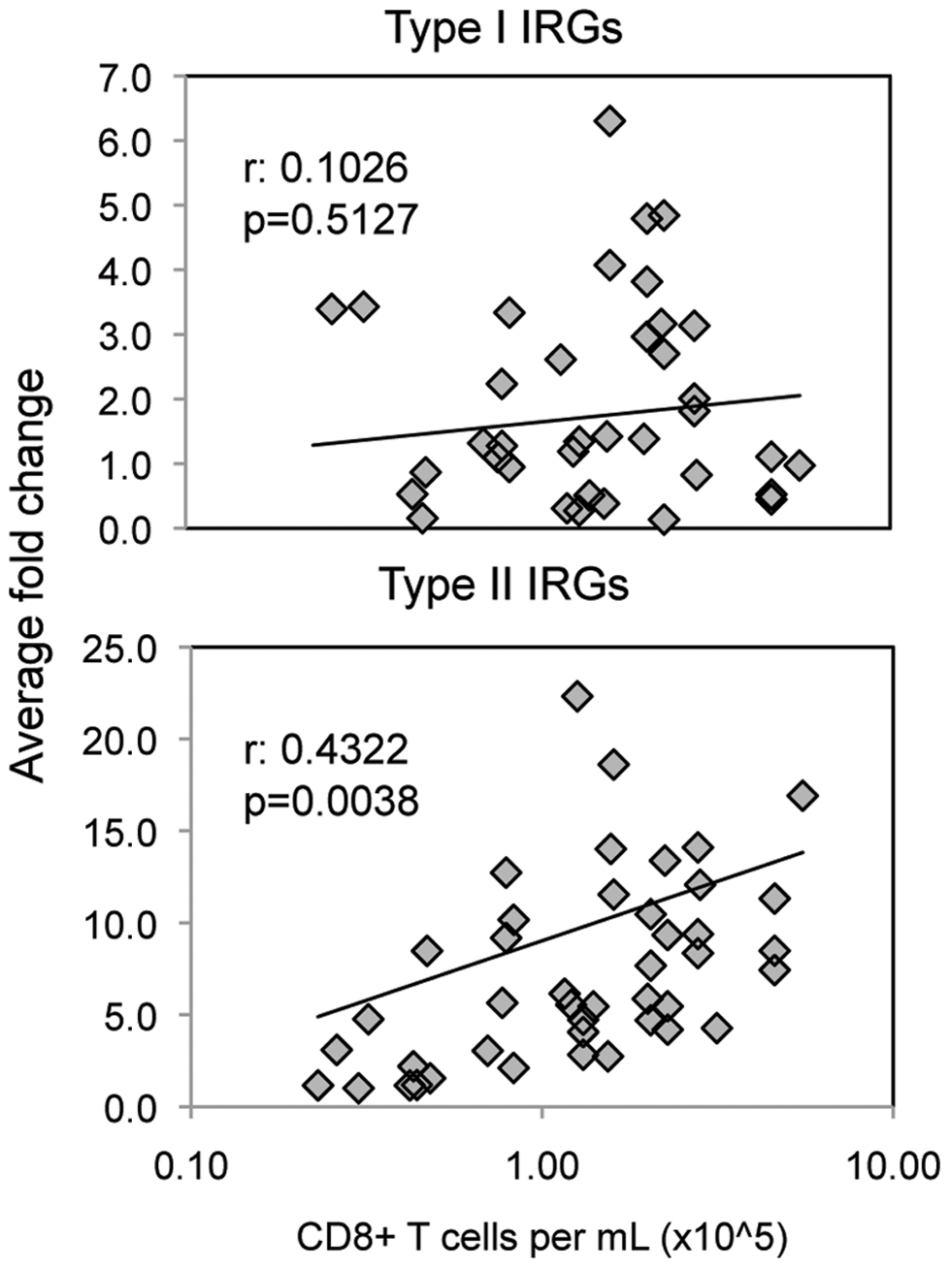
Type II IRG gene expression correlates with CD8 lymphocytosis in primary EBV infection. Data show the correlation between IRG gene expression and CD8 lymphocytosis in 42 subjects at timepoints within the first two weeks after symptom onset during primary EBV infection. The number of CD8 T cells per mL of peripheral blood was determined by flow cytometry (note: average at baseline was 0.25×10^6^). Superarray analysis by qPCR was performed to determine average fold changes for groups of Type I IRGs and Type II IRGs. Pearson correlation coefficients (r-values) and p-values are shown.

Finally, we investigated whether or not there was a correlation between gene expression and severity of illness during acute infection. A hallmark of infectious mononucleosis is the dramatic expansion of CD8 T cells. Indeed, both the total number of CD8 T cells/ml blood or the ratio of CD8 to CD4 T cells in the blood provided the best predictor of disease severity in a large prospective study. [38] Thus, using data from the qPCR superarray approach described above, we examined the correlation between gene expression and CD8 lymphocytosis. We found that expression of type I IRGs individually, or as a group, did not correlate strongly (Figure 7). This was true even when we limited the analysis to specific time windows where type I IRG gene expression was highest (data not shown). On the other hand, upregulation of type II IRG as a group did correlate positively (Figure 7). The upregulation of three individual genes correlated very significantly with CD8 lymphocytosis. These three: *OASL*, (Pearson r = 0.6059, p<0.0001) *TYMS* (r = 0.5019, p = 0.0006), and *SLAMF8* (r = 0.6028, p<0.0001), are all upregulated by IFNγ consistent with the known effects of IFNγ when given as therapy [39], [40]. Interestingly, *OASL* in particular has been hypothesized to cause fatigue through antagonism of the thyroid receptor [41]. Altogether these results reinforce the concept that many IM symptoms are immunopathologic, resulting from high levels of IFNγ produced by either innate or adaptive immune cells.

## Discussion

This study provides the first gene expression profiling of a natural herpes virus infection in healthy human subjects. One notable aspect of the EBV gene expression profile was the striking upregulation of genes related to cell cycle and proliferation. While proliferation of cells is likely to be part of the immune response to any pathogenic virus, what is striking is the magnitude and duration of these gene expression changes, which allow them to be observed at the level of whole blood gene expression profiling. Most other viral infections did not display significant upregulation of these genes at any time point. The exception was DENV. Indeed the shared profile of EBV and DENV was also observed in hemophagocytic syndromes, emphasizing the exaggerated inflammatory response that ensues in these viral infections. Although other potentially interesting comparisons would be to DNA viruses, particularly CMV, which can cause IM, there are currently no data available on gene expression during primary infection in humans.

In contrast to the readily apparent gene signature in acute infection, we were surprised to discover that there were no consistently detectable gene expression changes in human blood during latent infection, despite the sensitivity of our approach. EBV is known to persist in the B cell compartment, albeit at quite low levels (1–50 copies per million B cells). [42] The virus persists in a very “quiet” state, likely expressing viral RNAs (EBERs) but little or no viral protein products. [43] Nonetheless, immunosuppression can result in viral recrudescence with sometimes fatal consequences, [44], [45] thus EBV is presumed to be under constant active immune surveillance. However, EBV is unlikely to be the only chronic viral infection present in our subjects. Up to a dozen chronic viral infections can be common in humans, including the related herpesvirus CMV [46] although it should be noted that the majority of our subjects (88%) were CMV negative and remained so throughout the study.

Through comparison of the EBV signature to gene expression profiles of other acute viral infections in healthy individuals, we were able to identify the common and unique aspects of the response to EBV. While the response to all infections involved upregulation of IRGs, the response to EBV was notably lacking the upregulation of a subset of nine key IRGs. These particular genes are of interest as potential targets of viral antagonism. In addition we observed a striking downregulation of *IL1B*, which was surprising given that monocytes are activated and proliferating during acute EBV infection. Recent data suggested IL-1**β** may be downregulated by type I IFN in human monocytic cells *in vitro*. [47] However, it is not clear why *IL1B* would be downregulated by IFN in EBV but upregulated by IFN in most other viral infections. Another possibility was suggested by a recent report, which showed that EBV encodes a microRNA (BART15) that targets the NLRP3 inflammasome, and exosome mediated secretion of this miRNA resulted in downregulation of IL-1β in bystander (uninfected) monocytes *in vitro*. [48] Intriguingly, *IL1B* was also down regulated in most DENV patients and in about half of subjects given the YFV vaccine (Figure 3). This disparate regulation may be interesting in the context of monopoeisis and an environment of high IFNγ production. In patients administered G-CSF, immature monocytes entering the blood stream from the bone marrow bear surface bound IL-10. [49] In the presence of IL-10, monocytes that have become activated by IFNγ produce much less *IL1B*. [50] Furthermore, EBV encodes a viral IL-10 homologue (vIL-10) [51], suggesting another possible explanation for our observation. Further study will be required to determine the mechanism and significance of the profound *IL1B* downregulation. We hypothesize that the lack of upregulation of *IL1B* and key IRGs may allow this particular herpesvirus to avoid elicitation of adaptive immunity for many weeks, which may aid in establishing latency and provide an explanation for the long incubation period prior to symptoms.

## Supporting Information

Figure S1 **Comparison of fold changes obtained by qPCR and microarray.**
(PDF)

Figure S2 **EBV demonstrates a bias toward a type II IRG expression pattern.**
(PDF)

Figure S3 **Gene set analysis can successfully segregate the EBV signature from other acute illnesses.**
(PDF)

Figure S4 **Timecourse of gene changes during EBV infection compared to YFV, Poly IC, and DENV.**
(PDF)

Table S1 **Characteristics of the subjects chosen for microarray analysis.**
(DOCX)

Table S2 **List of significantly changed genes during acute IM.**
(DOCX)

Table S3 **Genes with a fold change ≥3.**
(DOCX)

Table S4 **EBV “unique” genes.**
(DOCX)
