# Supplementary material for: Primary EBV Infection Induces an Expression Profile Distinct from Other Viruses but Similar to Hemophagocytic Syndromes
Source: PLoS One. 2014 Jan 17;9(1):e85422. doi: 10.1371/journal.pone.0085422 (PMC3894977; doi:10.1371/journal.pone.0085422)
Supplement: Figure S1 — Comparison of fold changes obtained by qPCR and microarray. (PDF) [file pone.0085422.s001.pdf]

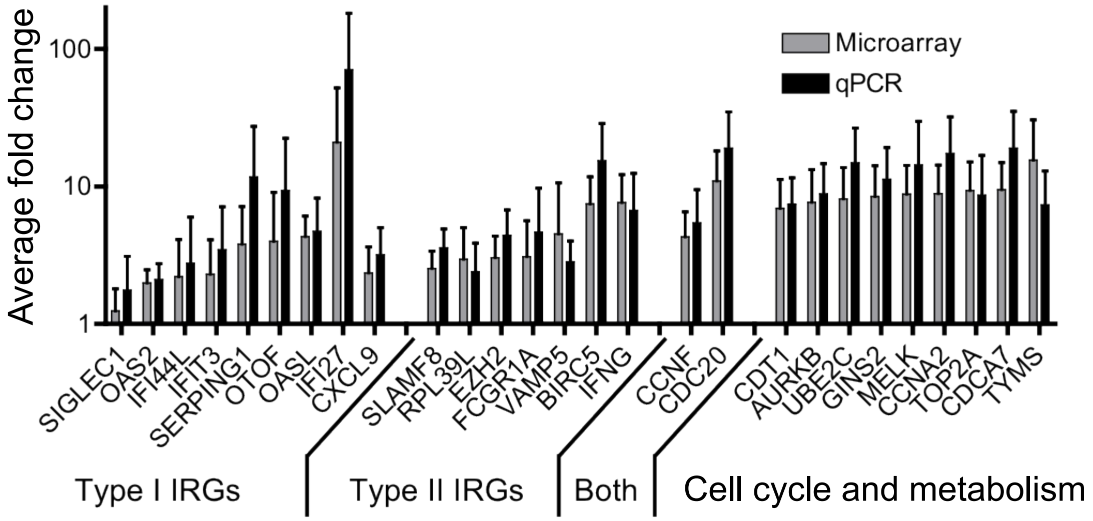

Figure S1: Comparison of fold changes obtained by qPCR and microarray

Average fold changes of eight patients between healthy baseline and acute sample are shown for all upregulated genes included on the qPCR array. Microarray values are shown in gray, qPCR values are shown in black. Error bars are +/- standard deviation.
