# Supplementary material for: Primary EBV Infection Induces an Expression Profile Distinct from Other Viruses but Similar to Hemophagocytic Syndromes
Source: PLoS One. 2014 Jan 17;9(1):e85422. doi: 10.1371/journal.pone.0085422 (PMC3894977; doi:10.1371/journal.pone.0085422)
Supplement: Figure S2 — EBV demonstrates a bias toward a type II IRG expression pattern. (PDF) [file pone.0085422.s002.pdf]

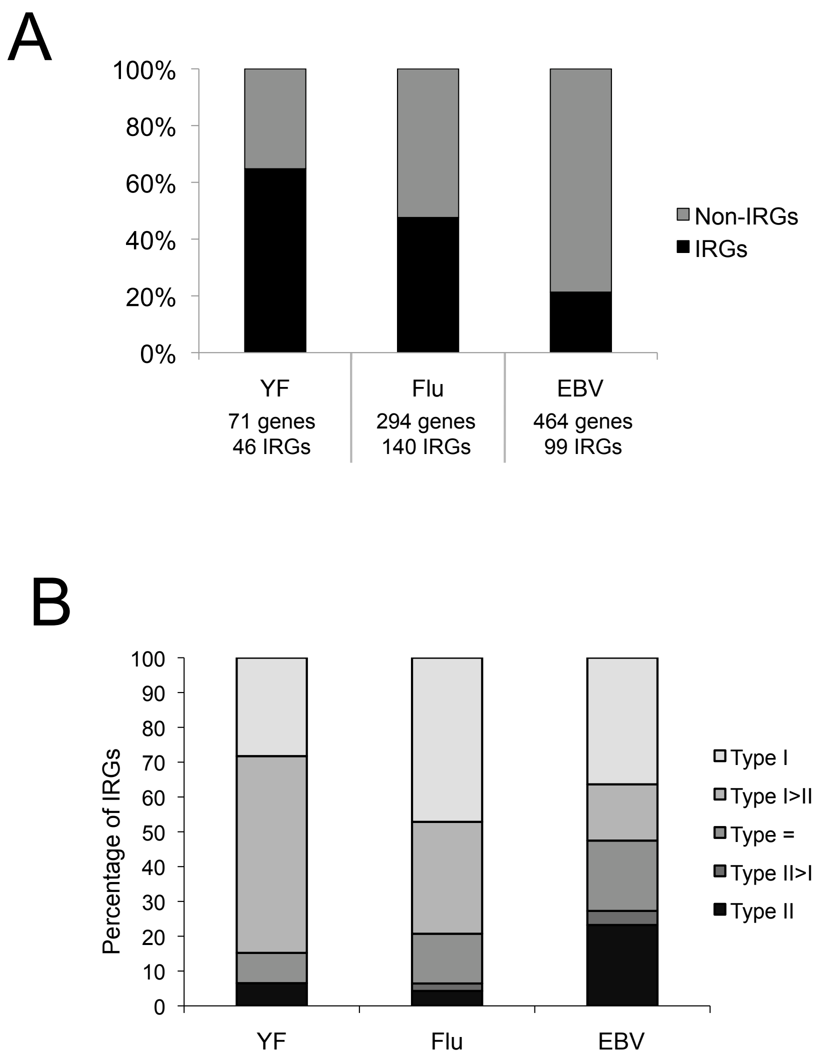

Figure S2: EBV demonstrates a bias toward a type II IRG expression pattern

(A) Genes upregulated during acute viral infection were divided into IRG and non-IRG subsets. The number of genes that changed significantly are listed for each infection. The proportion of IRG and non-IRG are shown in the bar graph. (B) Only genes classified as IRG were used for this analysis. IRGs were separated into groupings based on relative upregulation by type I and type II IFN in PBMC stimulated with IFN *in vitro*.
