# Supplementary material for: Primary EBV Infection Induces an Expression Profile Distinct from Other Viruses but Similar to Hemophagocytic Syndromes
Source: PLoS One. 2014 Jan 17;9(1):e85422. doi: 10.1371/journal.pone.0085422 (PMC3894977; doi:10.1371/journal.pone.0085422)
Supplement: Figure S3 — Gene set analysis can successfully segregate the EBV signature from other acute illnesses. (PDF) [file pone.0085422.s003.pdf]

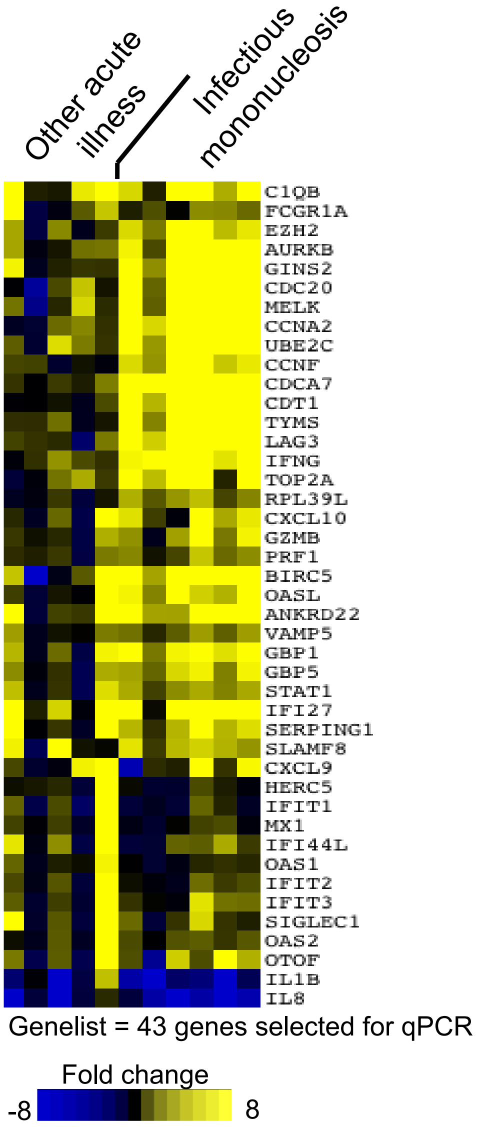


**Figure S3: Gene set analysis can successfully segregate the EBV signature from other acute illnesses.**

Gene expression of the indicated genes was determined by qPCR on 11 symptomatic subjects. Heat map indicated fold change from each subject's own baseline. K-means clustering was performed on 8 blinded samples and three known EBV samples. Analysis was performed with three clusters and 10,000 runs. Three samples were obtained from subjects presenting with acute primary EBV infection. Five additional samples were collected from subjects presenting with other undefined acute illnesses. The symptomatic primary EBV infection signature is characterized by high cell cycle gene expression, low type I IRG expression, and high *IFNG* expression.
