# Supplementary material for: Primary EBV Infection Induces an Expression Profile Distinct from Other Viruses but Similar to Hemophagocytic Syndromes
Source: PLoS One. 2014 Jan 17;9(1):e85422. doi: 10.1371/journal.pone.0085422 (PMC3894977; doi:10.1371/journal.pone.0085422)
Supplement: Figure S4 — Timecourse of gene changes during EBV infection compared to YFV, Poly IC, and DENV. (PDF) [file pone.0085422.s004.pdf]

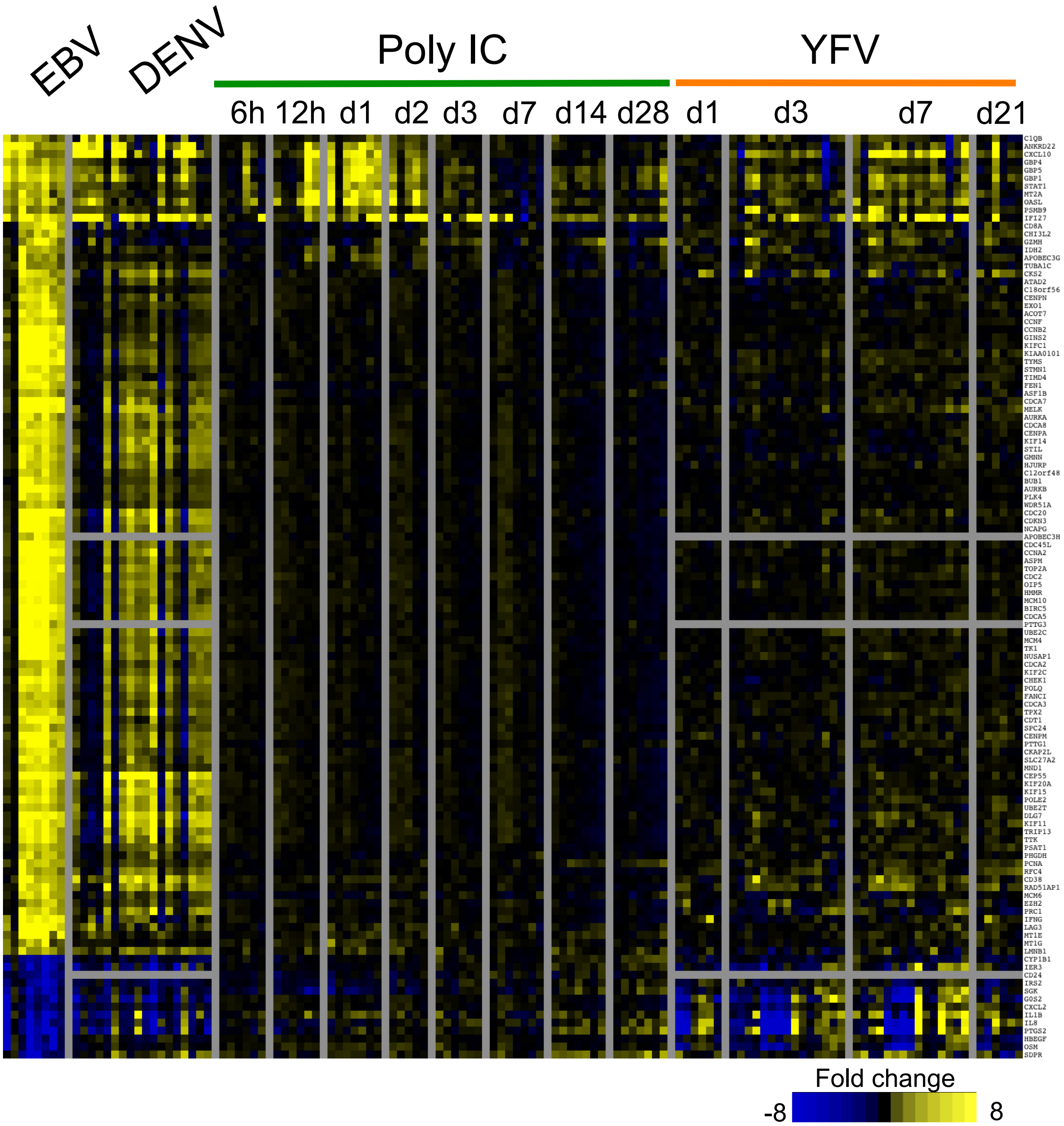

Figure S4: Most EBV/DENV induced genes were not increased at any timepoint following YFV vaccination or Poly IC treatment.

The genes shown were derived from analysis of EBV subjects (fold change ≥ 3 and met a statistical significance cutoff of p-value of ≤ 0.05 with Bonferonni multiple-tests correction). Gene expression is shown in comparison to subjects with other viral infections (YFV and DENV) or subjects injected with Poly IC. Data is arranged as a hierarchical clustering of genes. Each column represents a single subject. Horizontal gray bars indicate that the gene of interest was not present in the analyzed dataset used for comparison. Vertical gray bars separate disease or time groupings. The color intensity represents fold changes in gene expression in comparison to either each subjects' own healthy baseline (EBV, YFV and Poly IC) or in comparison to healthy controls (DENV).
