# Supplementary material for: Primary EBV Infection Induces an Expression Profile Distinct from Other Viruses but Similar to Hemophagocytic Syndromes
Source: PLoS One. 2014 Jan 17;9(1):e85422. doi: 10.1371/journal.pone.0085422 (PMC3894977; doi:10.1371/journal.pone.0085422)
Supplement: Table S1 — Characteristics of the subjects chosen for microarray analysis. (DOCX) [file pone.0085422.s005.docx]

**Table S1. Characteristics of the subjects chosen for microarray analysis**.

| Patient ID | |  | PRE |  | ACUTE |  | LATENT |
| --- | --- | --- | --- | --- | --- | --- | --- |
| 5027 | | Day* | -137 |  | -15 |  | 566 |
| SOI | 5 | VCA IgM# | (-) |  | - |  |  |
| Gender | Female | VCA IgG# | - |  | - |  | +++ |
| Duration | 42 days | Oral virus** | (-) |  | 800 |  | 11400 |
| CMV | neg | Blood virus** | (-) |  | - |  | - |
| 5088 | | Day | -118 |  | -4 |  | 406 |
| SOI | 4 | VCA IgM | (-) |  | - |  |  |
| Gender | Male | VCA IgG | - |  | - |  | +++ |
| Duration | 21 | Oral Virus | (-) |  | 15,000 |  | - |
| CMV | neg | Blood virus | (-) |  | - |  | - |
| 5509 | | Day | -119 |  | -4 |  | 563 |
| SOI | 3 | VCA IgM | (-) |  | - |  |  |
| Gender | Female | VCA IgG | - |  | - |  | (+++) |
| Duration | 8 days | Oral Virus | (-) |  | 42,500 |  | - |
| CMV | neg | Blood virus | (-) |  | 18,800 |  | - |
| 5342 | | Day | -115 |  | 0 |  | 704 |
| SOI | 4 | VCA IgM | (-) |  | + / - |  |  |
| Gender | Female | VCA IgG | - |  | - |  | +++ |
| Duration | 27 days | Oral Virus | (-) |  | 67,000 |  | 1300 |
| CMV | neg | Blood virus | (-) |  | 5,000 |  | - |
| 5483 | | Day | -97 |  | 3 |  | 231 |
| SOI | 3 | VCA IgM | (-) |  | +++ |  |  |
| Gender | Female | VCA IgG | - |  | - |  | +++ |
| Duration | 25 days | Oral Virus | (-) |  | 27,300 |  | - |
| CMV | neg | Blood virus | (-) |  | 4,400 |  | - |
| 5036 | | Day | -139 |  | 2 |  | 341 |
| SOI | 4 | VCA IgM | (-) |  | ++ |  |  |
| Gender | Male | VCA IgG | - |  | - |  | (++) |
| Duration | 19 days | Oral Virus | (-) |  | 1,000 |  | 4400 |
| CMV | neg | Blood virus | (-) |  | 11,400 |  | - |
| 5524 | | Day | -132 |  | 7 |  | 547 |
| SOI | 4 | VCA IgM | (-) |  | +++ |  |  |
| Gender | Female | VCA IgG | - |  | +++ |  | (+++) |
| Duration | 66 days | Oral Virus | (-) |  | 49,900 |  | - |
| CMV | pos | Blood virus | (-) |  | 60,000 |  | - |
| 5324 | | Day | -92 |  | 14 |  | 420 |
| SOI | 5 | VCA IgM | (-) |  | +++ |  |  |
| Gender | Male | VCA IgG | - |  | - |  | +++ |
| Duration | 15 days | Oral Virus | (-) |  | 2,500,000 |  | 1200 |
| CMV | pos | Blood virus | (-) |  | 200 |  | - |
| 5139 | | Day | -157 |  | No sample |  | 534 |
| SOI | 2 | VCA IgM | (-) |  |  |  |  |
| Gender | Male | VCA IgG | - |  |  |  | (+++) |
| Duration | 15 days | Oral Virus | (-) |  |  |  | - |
| CMV | pos | Blood virus | (-) |  |  |  | - |
| 5370 | | Day | -94 |  | No sample |  | 354 |
| SOI | 3 | VCA IgM | (-) |  |  |  |  |
| Gender | Female | VCA IgG | - |  |  |  | (+++) |
| Duration | ND | Oral Virus | (-) |  |  |  | 700 |
| CMV | neg | Blood virus | (-) |  |  |  | 200 |
|  |  | Average | -120 |  | 0 |  | 467 |

* *Day relative to symptom onset*

*# IgM or IgG antibodies to EBV viral capsid antigen*

*** Copies/mL of viral DNA detected in oral cells or whole blood*

*Values in parentheses are implied, based on values in adjacent timepoints (not shown)*

**Table S1.** EBV-naïve freshman were enrolled in a prospective clinical study and followed over the course of their undergraduate education. Samples were collected at multiple intervals before, during, and after primary infection with EBV. Of 66 subjects, 10 were selected for microarray analysis. Anti-EBV antibody titers, EBV viral loads, and other patient information for the “PRE” “ACUTE” and “LATENT” timepoints are shown. SOI, Severity of illness; Duration is days of symptoms.
