# Supplementary material for: Primary EBV Infection Induces an Expression Profile Distinct from Other Viruses but Similar to Hemophagocytic Syndromes
Source: PLoS One. 2014 Jan 17;9(1):e85422. doi: 10.1371/journal.pone.0085422 (PMC3894977; doi:10.1371/journal.pone.0085422)
Supplement: Table S2 — List of significantly changed genes during acute IM. (DOCX) [file pone.0085422.s006.docx]

**Table S2. List of genes significantly changed during acute IM**

| **Gene Symbol** | **Common and/or Official Name** | **Fold Change** |
| --- | --- | --- |
| IFI27 | interferon, alpha-inducible protein 27 | 10.416 |
| TYMS | TS or thymidylate synthetase | 8.973 |
| KIAA0101 | PAF, PAF15, p15PAF, or p15(PAF) | 8.162 |
| CDC20 | cell division cycle 20 homolog | 7.391 |
| CDCA7 | JPO1 or cell division cycle associated 7 | 6.864 |
| TOP2A | topoisomerase (DNA) II alpha | 6.692 |
| ANKRD22 | ankyrin repeat domain 22 | 6.680 |
| CCNB2 | cyclin B2 | 6.644 |
| MELK | maternal embryonic leucine zipper kinase | 6.491 |
| CCNA2 | cyclin A2 | 6.451 |
| GINS2 | GINS complex subunit 2 | 6.413 |
| GBP1 | guanylate binding protein 1, interferon-inducible | 6.396 |
| CDC45L | cell division cycle 45 homolog | 6.373 |
| CDCA5 | SORORIN or cell division cycle associated 5 | 6.267 |
| LAG3 | lymphocyte-activation gene 3 | 6.069 |
| MT1E | metallothionein 1E | 6.049 |
| IFNG | interferon, gamma | 5.876 |
| UBE2C | ubiquitin-conjugating enzyme E2C | 5.794 |
| TK1 | thymidine kinase 1, soluble | 5.669 |
| KIFC1 | kinesin family member C1 | 5.554 |
| BIRC5 | baculoviral IAP repeat containing 5 | 5.553 |
| MCM4 | minichromosome maintenance complex component 4 | 5.537 |
| CEP55 | centrosomal protein 55kDa | 5.532 |
| GBP5 | guanylate binding protein 5 | 5.441 |
| CDT1 | chromatin licensing and DNA replication factor 1 | 5.278 |
| AURKB | aurora kinase B | 5.263 |
| CDC2 | CDK1 or cyclin-dependent kinase 1 | 5.246 |
| MCM2 | minichromosome maintenance complex component 2 | 5.225 |
| CDKN3 | cyclin-dependent kinase inhibitor 3 or KAP1 | 5.170 |
| DLG7 | HURP or DLGAP5 | 5.162 |
| HMMR | RHAMM or hyaluronan-mediated motility receptor | 5.047 |
| ACOT7 | BACH or acyl-CoA thioesterase 7 | 5.018 |
| NCAPG | non-SMC condensin I complex, subunit G | 4.972 |
| NUSAP1 | nucleolar and spindle associated protein 1 | 4.965 |
| CHEK1 | checkpoint kinase 1 or CHK1 | 4.819 |
| MCM10 | minichromosome maintenance complex component 10 | 4.812 |
| OIP5 | LINT-25 | 4.774 |
| CENPM | centromere protein M | 4.772 |
| TIMD4 | TIM4 or T-cell immunoglobulin and mucin domain containing 4 | 4.765 |
| PTTG1 | pituitary tumor-transforming 1 | 4.720 |
| KIF11 | EG5 | 4.661 |
| ASPM | asp (abnormal spindle) homolog, microcephaly associated | 4.653 |
| KIF20A | MKLP2 | 4.584 |
| CXCL10 | IP-10 | 4.529 |
| PTTG3 | PTTG3P or pituitary tumor-transforming 3, pseudogene | 4.434 |
| STMN1 | stathmin 1 | 4.222 |
| FEN1 | flap structure-specific endonuclease 1 | 4.196 |
| POLE2 | polymerase (DNA directed), epsilon 2, accessory subunit | 4.169 |
| TRIP13 | thyroid hormone receptor interactor 13 | 4.130 |
| TTK | MPS1 | 4.075 |
| STAT1 | signal transducer and activator of transcription 1 | 4.036 |
| CENPN | centromere protein N | 3.982 |
| OASL | 2'-5'-oligoadenylate synthetase-like | 3.975 |
| TPX2 | microtubule-associated, homolog | 3.975 |
| CENPA | centromere protein A | 3.963 |
| GMNN | geminin, DNA replication inhibitor | 3.918 |
| PLK4 | polo-like kinase 4 or SAK | 3.909 |
| SLC27A2 | solute carrier family 27 (fatty acid transporter), member 2 | 3.862 |
| POLQ | polymerase (DNA directed), theta | 3.840 |
| CKAP2L | cytoskeleton associated protein 2-like | 3.833 |
| TUBA1C | tubulin, alpha 1c | 3.825 |
| CD38 | CD38 molecule | 3.823 |
| MT2A | metallothionein 2A | 3.823 |
| EXO1 | exonuclease 1 | 3.814 |
| RAD51AP1 | RAD51 associated protein 1 | 3.807 |
| GBP4 | guanylate binding protein 4 | 3.796 |
| BUB1 | budding uninhibited by benzimidazoles 1 | 3.754 |
| STIL | SCL/TAL1 interrupting locus or SIL | 3.721 |
| PCNA | proliferating cell nuclear antigen | 3.675 |
| HJURP | Holliday junction recognition protein | 3.671 |
| AURKA | aurora A or aurora kinase A | 3.669 |
| KIF14 | kinesin family member 14 | 3.659 |
| CD8A | CD8a molecule | 3.632 |
| APOBEC3H | apolipoprotein B mRNA editing enzyme, catalytic polypeptide-like 3H | 3.607 |
| MND1 | meiotic nuclear divisions 1 homolog | 3.602 |
| CDCA8 | BOREALIN or cell division cycle associated 8 | 3.596 |
| CKS2 | CDC28 protein kinase regulatory subunit 2 | 3.592 |
| PRC1 | protein regulator of cytokinesis 1 | 3.574 |
| MCM6 | minichromosome maintenance complex component 6 | 3.566 |
| CCNF | cyclin F or FBX1 | 3.553 |
| SPC24 | NDC80 kinetochore complex component, homolog | 3.417 |
| E2F2 | E2F transcription factor 2 | 3.413 |
| KIF15 | HKLP2 | 3.413 |
| PSMB9 | proteasome (prosome, macropain) subunit, beta type, 9 (large multifunctional peptidase 2) | 3.392 |
| C12orf48 | PARPBP; PARI | 3.391 |
| KIF2C | MCAK | 3.390 |
| C18orf56 | chromosome 18 open reading frame 56 | 3.389 |
| GZMH | granzyme H | 3.318 |
| IDH2 | isocitrate dehydrogenase 2 (NADP+), mitochondrial | 3.286 |
| CDCA2 | Repo-Man or cell division cycle associated 2 | 3.242 |
| FANCI | Fanconi anemia, complementation group I | 3.234 |
| ATAD2 | ANCCA or ATPase family, AAA domain containing 2 | 3.232 |
| UBE2T | ubiquitin-conjugating enzyme E2T | 3.214 |
| C1QB | complement component 1, q subcomponent, B chain | 3.208 |
| LMNB1 | lamin B1 | 3.168 |
| CDCA3 | cell division cycle associated 3 | 3.160 |
| ASF1B | ASF1 anti-silencing function 1 homolog B or CIA-II | 3.147 |
| APOBEC3G | apolipoprotein B mRNA editing enzyme, catalytic polypeptide-like 3G | 3.146 |
| PHGDH | phosphoglycerate dehydrogenase | 3.141 |
| RFC4 | replication factor C (activator 1) 4 | 3.111 |
| WDR51A | POC1A or PIX2 | 3.107 |
| MT1G | metallothionein 1G | 3.048 |
| PSAT1 | phosphoserine aminotransferase 1 | 3.040 |
| CHI3L2 | YKL-39 or chitinase 3-like 2 | 3.026 |
| C6orf173 | CENPW; CUG2; centromere protein W | 2.999 |
| NDC80 | HEC1; TID3; KNTC2 | 2.978 |
| MT1A | metallothionein 1A | 2.950 |
| C3orf14 | chromosome 3 open reading frame 14 | 2.937 |
| IL32 | interleukin 32 | 2.909 |
| VAMP5 | vesicle-associated membrane protein 5 | 2.907 |
| MCM5 | minichromosome maintenance complex component 5 | 2.905 |
| MTE | MT1IP; metallothionein 1I, pseudogene | 2.897 |
| FCGR1B | Fc fragment of IgG, high affinity Ib, receptor (CD64) | 2.876 |
| CHAF1B | CAF1; chromatin assembly factor 1, subunit B (p60) | 2.853 |
| SERPING1 | serpin peptidase inhibitor, clade G (C1 inhibitor), member 1 | 2.844 |
| HELLS | LSH; helicase, lymphoid-specific | 2.837 |
| RRM1 | ribonucleotide reductase M1 | 2.834 |
| RACGAP1 | MgcRacGAP; Rac GTPase activating protein 1 | 2.814 |
| SCD | SCD1; stearoyl-CoA desaturase (delta-9-desaturase) | 2.781 |
| PSME2 | PA28B; proteasome (prosome, macropain) activator subunit 2 (PA28 beta) | 2.769 |
| CDC7 | CDC7L1; cell division cycle 7 homolog (S. cerevisiae) | 2.767 |
| C16orf75 | RMI2, RecQ mediated genome instability 2, homolog (S. cerevisiae) | 2.747 |
| RAD51 | RAD51 homolog (S. cerevisiae) | 2.747 |
| SMC2 | CAP-E; structural maintenance of chromosomes 2 | 2.738 |
| FHL2 | four and a half LIM domains 2 | 2.724 |
| SMC4 | CAP-C; structural maintenance of chromosomes 4 | 2.692 |
| LY6E | RIGE; SCA2; TSA-1 | 2.682 |
| RBBP8 | CTIP; SCKL2; retinoblastoma binding protein 8 | 2.682 |
| EPSTI1 | BRESI1; epithelial stromal interaction 1 (breast) | 2.664 |
| EZH2 | enhancer of zeste homolog 2 (Drosophila) | 2.651 |
| RPL29 | HIP; L29; ribosomal protein L29 | 2.651 |
| HMGA1 | high mobility group AT-hook 1 | 2.636 |
| CD8B | CD8b molecule | 2.630 |
| C20orf100 | TOX2; TOX high mobility group box family member 2 | 2.628 |
| ENDOGL1 | EXOG; endo/exonuclease (5'-3'), endonuclease G-like | 2.618 |
| TIMELESS | TIM; timeless homolog (Drosophila) | 2.618 |
| C18orf24 | SKA1; spindle and kinetochore associated complex subunit 1 | 2.610 |
| FBXO6 | FBS2; F-box protein 6 | 2.601 |
| MYL6B | myosin, light chain 6B, alkali, smooth muscle and non-muscle | 2.588 |
| USP54 | ubiquitin specific peptidase 54 | 2.587 |
| FLJ33590 | CXXC11; CXXC finger protein 11 | 2.583 |
| PYHIN1 | IFIX; pyrin and HIN domain family, member 1 | 2.564 |
| RARRES3 | RIG1; TIG3; retinoic acid receptor responder (tazarotene induced) 3 | 2.560 |
| LAP3 | leucine aminopeptidase 3 | 2.543 |
| TROAP | TASTIN; trophinin associated protein | 2.543 |
| GFI1 | SCN2; growth factor independent 1 transcription repressor | 2.540 |
| PSMB8 | LMP7; proteasome (prosome, macropain) subunit, beta type, 8 (large multifunctional peptidase 7) | 2.524 |
| MCM7 | minichromosome maintenance complex component 7 | 2.522 |
| GCH1 | GTP cyclohydrolase 1 | 2.521 |
| TUBG1 | tubulin, gamma 1 | 2.516 |
| GALM | galactose mutarotase (aldose 1-epimerase) | 2.500 |
| DONSON | CIITA; downstream neighbor of SON | 2.487 |
| MTHFD2 | methylenetetrahydrofolate dehydrogenase (NADP+ dependent) 2, methenyltetrahydrofolate cyclohydrolase | 2.487 |
| MLF1IP | CENPU; MLF1 interacting protein | 2.486 |
| C17orf53 | chromosome 17 open reading frame 53 | 2.485 |
| GINS3 | PSF3; GINS complex subunit 3 (Psf3 homolog) | 2.485 |
| IFI6 | G1P3; interferon, alpha-inducible protein 6 | 2.483 |
| C16orf59 | chromosome 16 open reading frame 59 | 2.480 |
| GBP2 | guanylate binding protein 2, interferon-inducible | 2.476 |
| NME1 | NM23; NM23-H1; NME/NM23 nucleoside diphosphate kinase 1 | 2.464 |
| KLRD1 | CD94; killer cell lectin-like receptor subfamily D, member 1 | 2.460 |
| CDK2 | cyclin-dependent kinase 2 | 2.457 |
| TAP1 | transporter 1, ATP-binding cassette, sub-family B (MDR/TAP) | 2.452 |
| PRR11 | proline rich 11 | 2.447 |
| PSMA3 | HC8; proteasome (prosome, macropain) subunit, alpha type, 3 | 2.447 |
| OBFC2B | NABP2; SSB1; OBFC2B; nucleic acid binding protein 2 | 2.443 |
| C1orf41 | HSPB11; heat shock protein family B (small), member 11 | 2.437 |
| OTOF | DFNB9; DFNB6; otoferlin | 2.437 |
| RPL39L | ribosomal protein L39-like | 2.436 |
| UBE2L6 | UBCH8; ubiquitin-conjugating enzyme E2L 6 | 2.436 |
| MCM3 | minichromosome maintenance complex component 3 | 2.433 |
| ADA | adenosine deaminase | 2.429 |
| CACYBP | SIP; calcyclin binding protein | 2.429 |
| C6orf190 | THEMIS; thymocyte selection associated | 2.428 |
| CKS1B | CKS1; CDC28 protein kinase regulatory subunit 1B | 2.427 |
| SLAMF8 | SLAM family member 8 | 2.420 |
| FCGR1A | Fc fragment of IgG, high affinity Ia, receptor (CD64) | 2.414 |
| ZWILCH | Zwilch, kinetochore associated, homolog (Drosophila) | 2.412 |
| GTSE1 | G-2 and S-phase expressed 1 | 2.409 |
| NUDT1 | MTH1; nudix (nucleoside diphosphate linked moiety X)-type motif 1 | 2.408 |
| ZNF683 | zinc finger protein 683 | 2.396 |
| PPIL5 | LRR1; leucine rich repeat protein 1 | 2.387 |
| STK39 | SPAK; serine threonine kinase 39 | 2.377 |
| WARS | tryptophanyl-tRNA synthetase | 2.376 |
| TFDP1 | transcription factor Dp-1 | 2.369 |
| PAICS | ADE2; AIRC; PAIS; ADE2H1 | 2.362 |
| ANLN | anillin, actin binding protein | 2.359 |
| DUT | dUTPase; deoxyuridine triphosphatase | 2.356 |
| PDCD1 | PD-1; programmed cell death 1 | 2.344 |
| C6orf129 | CCDC167; coiled-coil domain containing 167 | 2.343 |
| PRF1 | perforin 1 (pore forming protein) | 2.335 |
| CASP7 | caspase 7, apoptosis-related cysteine peptidase | 2.330 |
| IFI16 | PYHIN2; interferon, gamma-inducible protein 16 | 2.324 |
| CCNE1 | cyclin E1 | 2.323 |
| PSMB2 | proteasome (prosome, macropain) subunit, beta type, 2 | 2.323 |
| CCDC28B | coiled-coil domain containing 28B | 2.318 |
| FGFBP2 | fibroblast growth factor binding protein 2 | 2.313 |
| HMGB2 | high mobility group box 2 | 2.313 |
| BRCA1 | breast cancer 1, early onset | 2.308 |
| GCUD2 | FAM72D; family with sequence similarity 72, member D | 2.299 |
| TIPIN | TIMELESS interacting protein | 2.293 |
| C16orf33 | SNRNP25; small nuclear ribonucleoprotein 25kDa (U11/U12) | 2.287 |
| WDR34 | WD repeat domain 34 | 2.287 |
| TUBB | tubulin, beta class I | 2.286 |
| RTP4 | IFRG28; receptor (chemosensory) transporter protein 4 | 2.275 |
| SLAMF7 | CS1; CD319; CRACC | 2.273 |
| MCOLN2 | TRPML2; mucolipin 2 | 2.266 |
| CCDC99 | SPDL1; spindle apparatus coiled-coil protein 1 | 2.262 |
| CCDC34 | coiled-coil domain containing 34 | 2.261 |
| FCRL6 | Fc receptor-like 6 | 2.261 |
| PARP9 | BAL1; poly (ADP-ribose) polymerase family, member 9 | 2.260 |
| RFC5 | RFC36; replication factor C (activator 1) 5, 36.5kDa | 2.259 |
| PSMG1 | PAC1; DSCR2; proteasome (prosome, macropain) assembly chaperone 1 | 2.252 |
| C1orf135 | AUNIP; AIBP; aurora kinase A and ninein interacting protein | 2.251 |
| APOBEC3B | apolipoprotein B mRNA editing enzyme, catalytic polypeptide-like 3B | 2.249 |
| ORC1L | ORC1; PARC1; origin recognition complex, subunit 1 | 2.247 |
| PHF19 | PCL3; PHD finger protein 19 | 2.247 |
| C9orf140 | SAPCD2; suppressor APC domain containing 2 | 2.243 |
| CENPL | centromere protein L | 2.239 |
| NUF2 | CDCA1; NUF2, NDC80 kinetochore complex component, homolog (S. cerevisiae) | 2.232 |
| IL28RA | interleukin 28 receptor, alpha (interferon, lambda receptor) | 2.222 |
| PXMP2 | PMP22; peroxisomal membrane protein 2, 22kDa | 2.218 |
| RANBP1 | RAN binding protein 1 | 2.218 |
| FASLG | FASL; CD178; CD95L; TNFSF6 | 2.216 |
| EBP | emopamil binding protein | 2.210 |
| PGAM1 | phosphoglycerate mutase 1 (brain) | 2.209 |
| ALS2CR4 | TMEM237 | 2.206 |
| TAP2 | transporter 2, ATP-binding cassette, sub-family B (MDR/TAP) | 2.205 |
| MRPL10 | mitochondrial ribosomal protein L10 | 2.200 |
| SH2D1A | LYP; SAP; XLP; SH2 domain containing 1A | 2.199 |
| IFIT3 | IFIT4; RIG-G; interferon-induced protein with tetratricopeptide repeats 3 | 2.197 |
| ARPC5L | ctin related protein 2/3 complex, subunit 5-like | 2.196 |
| TIPRL | TIP41, TOR signaling pathway regulator-like (S. cerevisiae) | 2.194 |
| POLA2 | polymerase (DNA directed), alpha 2, accessory subunit | 2.187 |
| UNG | UNG1; UNG2; uracil-DNA glycosylase | 2.186 |
| LGALS3BP | MAC-2-BP; G3BP; lectin, galactoside-binding, soluble, 3 binding protein | 2.184 |
| RPA3 | replication protein A3, 14kDa | 2.177 |
| SNRPA | U1A; small nuclear ribonucleoprotein polypeptide A | 2.177 |
| BAK1 | BAK; BCL2-antagonist/killer 1 | 2.176 |
| APOL3 | apolipoprotein L, 3 | 2.173 |
| TCTEX1D2 | Tctex1 domain containing 2 | 2.173 |
| NCAPD2 | CNAP1; non-SMC condensin I complex, subunit D2 | 2.168 |
| GZMB | granzyme B (granzyme 2, cytotoxic T-lymphocyte-associated serine esterase 1) | 2.167 |
| APITD1 | MHF1; CENPS | 2.165 |
| RPS7 | S7; ribosomal protein S7 | 2.165 |
| FAM33A | SKA2; spindle and kinetochore associated complex subunit 2 | 2.164 |
| AGK | MULK; acylglycerol kinase | 2.156 |
| PTPLAD1 | B-IND1; protein tyrosine phosphatase-like A domain containing 1 | 2.154 |
| MRPL12 | mitochondrial ribosomal protein L12 | 2.153 |
| NUP37 | nucleoporin 37kDa | 2.153 |
| PRIM1 | primase, DNA, polypeptide 1 (49kDa) | 2.145 |
| DNAJC9 | DnaJ (Hsp40) homolog, subfamily C, member 9 | 2.134 |
| CENPK | centromere protein K | 2.131 |
| MTHFD1 | methylenetetrahydrofolate dehydrogenase (NADP+ dependent) 1, methenyltetrahydrofolate cyclohydrolase, formyltetrahydrofolate synthetase | 2.131 |
| KIF19 | kinesin family member 19 | 2.129 |
| CXCR3 | MigR; IP10-R; chemokine (C-X-C motif) receptor 3 | 2.128 |
| RAB33A | RabS10; RAB33A, member RAS oncogene family | 2.124 |
| FANCG | Fanconi anemia, complementation group G | 2.123 |
| GZMA | granzyme A (granzyme 1, cytotoxic T-lymphocyte-associated serine esterase 3) | 2.122 |
| CXCL9 | MIG; chemokine (C-X-C motif) ligand 9 | 2.120 |
| ISG15 | ISG15 ubiquitin-like modifier | 2.115 |
| XTP3TPA | DCTPP1; DCTPP1 dCTP pyrophosphatase 1 | 2.115 |
| MT1X | metallothionein 1X | 2.111 |
| PIF1 | PIF1 5'-to-3' DNA helicase homolog (S. cerevisiae) | 2.109 |
| CDC42 | cell division cycle 42 (GTP binding protein, 25kDa) | 2.107 |
| SAMD3 | sterile alpha motif domain containing 3 | 2.102 |
| TRMT5 | TRM5; tRNA methyltransferase 5 homolog (S. cerevisiae) | 2.101 |
| KEAP1 | kelch-like ECH-associated protein 1 | 2.099 |
| PTPN7 | HEPTP; protein tyrosine phosphatase, non-receptor type 7 | 2.099 |
| HMGN2 | high mobility group nucleosomal binding domain 2 | 2.087 |
| GART | phosphoribosylglycinamide formyltransferase, phosphoribosylglycinamide synthetase, phosphoribosylaminoimidazole synthetase | 2.082 |
| PAQR4 | progestin and adipoQ receptor family member IV | 2.078 |
| PSMA5 | PSC5; proteasome (prosome, macropain) subunit, alpha type, 5 | 2.078 |
| RALY | RNA binding protein, autoantigenic (hnRNP-associated with lethal yellow homolog (mouse)) | 2.078 |
| RAD54L | hRAD54; RAD54-like (S. cerevisiae) | 2.077 |
| TGFBR3 | betaglycan; transforming growth factor, beta receptor III | 2.076 |
| SLBP | stem-loop binding protein | 2.071 |
| KCNK10 | TREK-2; potassium channel, subfamily K, member 10 | 2.070 |
| MTHFD1L | methylenetetrahydrofolate dehydrogenase (NADP+ dependent) 1-like | 2.065 |
| RFC3 | RFC38; replication factor C (activator 1) 3, 38kDa | 2.065 |
| MSH6 | mutS homolog 6 (E. coli) | 2.063 |
| LIMA1 | EPLIN; LIM domain and actin binding 1 | 2.062 |
| UCK2 | UK; uridine-cytidine kinase 2 | 2.059 |
| GINS4 | GINS complex subunit 4 (Sld5 homolog) | 2.058 |
| DDX39 | URH49; DEAD (Asp-Glu-Ala-Asp) box polypeptide 39A | 2.056 |
| EXOSC3 | exosome component 3 | 2.056 |
| C1orf112 | chromosome 1 open reading frame 112 | 2.047 |
| POLA1 | polymerase (DNA directed), alpha 1, catalytic subunit | 2.044 |
| SPON2 | spondin 2, extracellular matrix protein | 2.044 |
| CASP3 | caspase 3, apoptosis-related cysteine peptidase | 2.043 |
| MT1F | metallothionein 1F | 2.040 |
| DPP3 | DPPIII; dipeptidyl-peptidase 3 | 2.039 |
| CBX5 | HP1; HP1A; chromobox homolog 5 | 2.038 |
| FBXO5 | F-box protein 5 | 2.036 |
| TXNL2 | GLRX3; PICOT | 2.036 |
| BLM | Bloom syndrome, RecQ helicase-like | 2.034 |
| INCENP | inner centromere protein antigens 135/155kDa | 2.034 |
| DCLRE1A | SNM1; SNM1A; DNA cross-link repair 1A | 2.031 |
| IFI35 | IFP35; interferon-induced protein 35 | 2.031 |
| CTPS | CTPS1; CTP synthase 1 | 2.030 |
| DSN1 | KNL3; MIS13; DSN1, MIND kinetochore complex component, homolog (S. cerevisiae) | 2.030 |
| MRPL22 | mitochondrial ribosomal protein L22 | 2.026 |
| PPP1CA | PP-1A; protein phosphatase 1, catalytic subunit, alpha isozyme | 2.022 |
| CDC123 | cell division cycle 123 homolog (S. cerevisiae) | 2.019 |
| VDAC1 | voltage-dependent anion channel 1 | 2.018 |
| PSMA4 | HC9; PSC9; proteasome (prosome, macropain) subunit, alpha type, 4 | 2.015 |
| C7orf24 | GGCT; CRF21; gamma-glutamylcyclotransferase | 2.009 |
| KNTC1 | ROD; kinetochore associated 1 | 2.009 |
| EIF2B2 | eukaryotic translation initiation factor 2B, subunit 2 beta, 39kDa | 2.008 |
| MRPL39 | mitochondrial ribosomal protein L39 | 2.008 |
| SIRPG | signal-regulatory protein gamma | 2.008 |
| TUBB2C | tubulin, beta 4B class IVb | 2.008 |
| TMEM97 | MAC30; transmembrane protein 97 | 2.007 |
| ACTL6A | BAF53A; actin-like 6A | 2.006 |
| SUV39H1 | suppressor of variegation 3-9 homolog 1 (Drosophila) | 2.006 |
| BATF | basic leucine zipper transcription factor, ATF-like | 2.004 |
| MIB2 | mindbomb E3 ubiquitin protein ligase 2 | 2.003 |
| GPR56 | G protein-coupled receptor 56 | 2.000 |
| TPPP3 | tubulin polymerization-promoting protein family member 3 | -2.000 |
| SWAP70 | SWAP switching B-cell complex 70kDa subunit | -2.001 |
| PLCB1 | phospholipase C, beta 1 (phosphoinositide-specific) | -2.003 |
| RNF130 | GOLIATH; ring finger protein 130 | -2.013 |
| CD300LB | CLM7; TREM5; IREM3 | -2.015 |
| TLR10 | toll-like receptor 10 | -2.016 |
| LAMC1 | laminin, gamma 1 (formerly LAMB2) | -2.020 |
| FCAR | CD89; Fc fragment of IgA, receptor for | -2.021 |
| VCAN | versican | -2.021 |
| LRP3 | low density lipoprotein receptor-related protein 3 | -2.026 |
| AFF3 | LAF4; AF4/FMR2 family, member 3 | -2.029 |
| TNFRSF4 | OX40; CD134 | -2.034 |
| ZNF395 | PBF; HDBP2 | -2.036 |
| CFD | DF; FD; complement factor D (adipsin) | -2.042 |
| GNG7 | guanine nucleotide binding protein (G protein), gamma 7 | -2.043 |
| RAB31 | Rab22B; RAB31, member RAS oncogene family | -2.043 |
| MEF2C | myocyte enhancer factor 2C | -2.044 |
| VWF | von Willebrand factor | -2.048 |
| CBX7 | chromobox homolog 7 | -2.056 |
| GALNAC4S-6ST | CHST15; BRAG; carbohydrate (N-acetylgalactosamine 4-sulfate 6-O) sulfotransferase 15 | -2.057 |
| S100A12 | ENRAGE; S100 calcium binding protein A12 | -2.059 |
| TSPAN9 | tetraspanin 9 | -2.061 |
| TCEA3 | TFIIS; transcription elongation factor A (SII), 3 | -2.065 |
| RASD1 | AGS1; DEXRAS1; RAS, dexamethasone-induced 1 | -2.068 |
| MPL | TPOR; myeloproliferative leukemia virus oncogene | -2.074 |
| PROK2 | BV8; prokineticin 2 | -2.077 |
| GPR162 | G protein-coupled receptor 162 | -2.079 |
| CCR7 | chemokine (C-C motif) receptor 7 | -2.080 |
| SSBP2 | single-stranded DNA binding protein 2 | -2.081 |
| TRIB1 | tribbles homolog 1 (Drosophila) | -2.084 |
| ZFHX3 | ATBF1; zinc finger homeobox 3 | -2.089 |
| CD79B | IGB; CD79b molecule, immunoglobulin-associated beta | -2.093 |
| BLK | B lymphoid tyrosine kinase | -2.095 |
| SLC26A11 | solute carrier family 26, member 11 | -2.095 |
| DENND2D | DENN/MADD domain containing 2D | -2.096 |
| PTCRA | pre T-cell antigen receptor alpha | -2.098 |
| ANG | angiogenin, ribonuclease, RNase A family, 5 | -2.106 |
| CXCL16 | SRPSOX; chemokine (C-X-C motif) ligand 16 | -2.110 |
| SPINT2 | HAI-2; serine peptidase inhibitor, Kunitz type, 2 | -2.112 |
| DAB2 | disabled homolog 2 | -2.121 |
| NUAK2 | SNARK; NUAK family, SNF1-like kinase, 2 | -2.123 |
| ITGB5 | integrin, beta 5 | -2.124 |
| TCEAL3 | transcription elongation factor A (SII)-like 3 | -2.127 |
| MOSC1 | MARC1; mitochondrial amidoxime reducing component 1 | -2.130 |
| RHOB | ras homolog family member B | -2.132 |
| LOC90925 | IGHV5-78; immunoglobulin heavy variable 5-78 (pseudogene) | -2.134 |
| LRRC26 | CAPC; leucine rich repeat containing 26 | -2.136 |
| MGC13057 | chromosome 2 open reading frame 88 | -2.136 |
| SOCS2 | suppressor of cytokine signaling 2 | -2.136 |
| MERTK | c-mer proto-oncogene tyrosine kinase | -2.137 |
| ACTN1 | actinin, alpha 1 | -2.138 |
| DDIT4 | Dig2; REDD1; DNA-damage-inducible transcript 4 | -2.146 |
| ZNF467 | EZI; zinc finger protein 467 | -2.156 |
| D4S234E | NSG1; NEEP21; neuron specific gene family member 1 | -2.157 |
| MGST1 | microsomal glutathione S-transferase 1 | -2.160 |
| VNN3 | vanin 3 | -2.161 |
| EGR2 | early growth response 2 | -2.172 |
| FLJ43093 | RAB44; RASD3; RASL13; member RAS oncogene family | -2.172 |
| DPEP2 | dipeptidase 2 | -2.183 |
| RTN1 | reticulon 1 | -2.190 |
| SVIL | supervillin | -2.194 |
| DEF8 | differentially expressed in FDCP 8 homolog (mouse) | -2.196 |
| GPR177 | EVI; MRP; GPR177 | -2.196 |
| FCRL2 | Fc receptor-like 2 | -2.198 |
| NELL2 | NEL-like 2 (chicken) | -2.201 |
| TBC1D9 | MDR1; TBC1 domain family, member 9 (with GRAM domain) | -2.227 |
| FAM134B | JK1; family with sequence similarity 134, member B | -2.233 |
| CD163 | CD163 molecule | -2.236 |
| IL11RA | interleukin 11 receptor, alpha | -2.236 |
| CD93 | C1qRP;CD93 molecule | -2.247 |
| SPRED1 | sprouty-related, EVH1 domain containing 1 | -2.248 |
| RGS2 | regulator of G-protein signaling 2, 24kDa | -2.250 |
| PDE5A | CN5A; PDE5; phosphodiesterase 5A, cGMP-specific | -2.251 |
| TGFBI | transforming growth factor, beta-induced, 68kDa | -2.253 |
| CLEC4A | DCIR; C-type lectin domain family 4, member A | -2.259 |
| C7orf41 | chromosome 7 open reading frame 41 | -2.268 |
| MS4A6A | membrane-spanning 4-domains, subfamily A, member 6A | -2.273 |
| TPM2 | tropomyosin 2 (beta) | -2.273 |
| RBP7 | CRBPIV; retinol binding protein 7, cellular | -2.283 |
| PTGDS | PGDS; LPGDS; prostaglandin D2 synthase 21kDa (brain) | -2.288 |
| CD19 | CD19 molecule | -2.290 |
| CXCR4 | chemokine (C-X-C motif) receptor 4 | -2.292 |
| RXRA | retinoid X receptor, alpha | -2.297 |
| BASP1 | brain abundant, membrane attached signal protein 1 | -2.308 |
| CACNA1I | Cav3.3; calcium channel, voltage-dependent, T type, alpha 1I subunit | -2.315 |
| COBLL1 | COBL-like 1 | -2.335 |
| NLRP12 | NALP12; NLR family, pyrin domain containing 12 | -2.336 |
| TSPAN18 | tetraspanin 18 | -2.343 |
| NR4A3 | nuclear receptor subfamily 4, group A, member 3 | -2.344 |
| KBTBD11 | kelch repeat and BTB (POZ) domain containing 11 | -2.349 |
| BCL11A | B-cell CLL/lymphoma 11A (zinc finger protein) | -2.350 |
| LTB | lymphotoxin beta (TNF superfamily, member 3) | -2.366 |
| CSF3R | CD114; GCSFR; colony stimulating factor 3 receptor (granulocyte) | -2.367 |
| MAL | mal, T-cell differentiation protein | -2.371 |
| FCGBP | Fc fragment of IgG binding protein | -2.376 |
| RNASE4 | ribonuclease, RNase A family, 4 | -2.386 |
| AKR1C3 | aldo-keto reductase family 1, member C3 | -2.396 |
| LRRN3 | NLRR3; leucine rich repeat neuronal 3 | -2.397 |
| VENTX | VENT homeobox | -2.397 |
| CXCR5 | chemokine (C-X-C motif) receptor 5 | -2.405 |
| CNTNAP2 | contactin associated protein-like 2 | -2.417 |
| ZSCAN18 | zinc finger and SCAN domain containing 18 | -2.449 |
| BANK1 | B-cell scaffold protein with ankyrin repeats 1 | -2.451 |
| C5orf29 | GAPT; GRB2-binding adaptor protein, transmembrane | -2.496 |
| TCL1A | TCL1; T-cell leukemia/lymphoma 1A | -2.503 |
| CYBRD1 | cytochrome b reductase 1 | -2.508 |
| ZDHHC1 | zinc finger, DHHC-type containing 1 | -2.512 |
| BCL2 | B-cell CLL/lymphoma 2 | -2.513 |
| LY86 | MD-1; lymphocyte antigen 86 | -2.523 |
| PDK4 | pyruvate dehydrogenase kinase, isozyme 4 | -2.527 |
| OSBPL10 | ORP10; OSBP9; oxysterol binding protein-like 10 | -2.529 |
| AXIN2 | axin 2 | -2.543 |
| VIPR1 | VPAC1; vasoactive intestinal peptide receptor 1 | -2.550 |
| CHST13 | C4ST3; carbohydrate (chondroitin 4) sulfotransferase 13 | -2.553 |
| CD83 | CD83 molecule | -2.570 |
| RGS18 | regulator of G-protein signaling 18 | -2.572 |
| SLC40A1 | FPN1; solute carrier family 40 (iron-regulated transporter), member 1 | -2.601 |
| VPREB3 | pre-B lymphocyte 3 | -2.606 |
| F13A1 | coagulation factor XIII, A1 polypeptide | -2.651 |
| CD79A | MB-1; IGA; CD79a molecule, immunoglobulin-associated alpha | -2.713 |
| FCRLA | FREB; Fc receptor-like A | -2.716 |
| PTGS1 | COX1; prostaglandin-endoperoxide synthase 1 (prostaglandin G/H synthase and cyclooxygenase) | -2.721 |
| PPBP | CXCL7; Beta-TG; NAP-2 | -2.747 |
| SH3BGRL2 | SH3 domain binding glutamic acid-rich protein like 2 | -2.754 |
| PADI4 | PAD4; peptidyl arginine deiminase, type IV | -2.836 |
| EBI2 | GPR183; G protein-coupled receptor 183 | -2.850 |
| C5AR1 | C5A; C5AR; CD88; complement component 5a receptor 1 | -2.873 |
| THBS1 | TSP1; thrombospondin 1 | -2.882 |
| RNASE6 | ribonuclease, RNase A family, k6 | -2.896 |
| GP9 | GPIX; glycoprotein IX (platelet) | -2.923 |
| NR4A2 | NURR1; nuclear receptor subfamily 4, group A, member 2 | -2.954 |
| CD1C | BDCA1; CD1c molecule | -2.959 |
| CACNA2D3 | calcium channel, voltage-dependent, alpha 2/delta subunit 3 | -3.000 |
| CD24 | CD24 molecule | -3.025 |
| CXCL2 | MIP-2 | -3.045 |
| G0S2 | G0/G1switch 2 | -3.059 |
| SGK | serum/glucocorticoid regulated kinase 1 | -3.260 |
| SDPR | serum deprivation response | -3.273 |
| CYP1B1 | cytochrome P450, family 1, subfamily B, polypeptide 1 | -3.480 |
| IRS2 | insulin receptor substrate 2 | -3.557 |
| HBEGF | heparin-binding EGF-like growth factor | -3.833 |
| OSM | oncostatin M | -4.324 |
| IER3 | IEX1 or immediate early response gene 3 | -4.398 |
| IL1B | Interleukin – 1beta | -4.520 |
| IL8 | Interleukin - 8 | -5.197 |
| PTGS2 | COX2 | -5.360 |

**Table S2.** Shows the complete list of 464 genes significantly up/down regulated during acute infection with EBV, the common and/or official name of the gene, as well as the average fold change within the EBV cohort. The genes shown were derived from analysis of EBV subjects (fold change ≥ 2 and met a statistical significance cutoff of p-value of ≤0.05 with Bonferonni multiple-tests correction).
