# Supplementary material for: Primary EBV Infection Induces an Expression Profile Distinct from Other Viruses but Similar to Hemophagocytic Syndromes
Source: PLoS One. 2014 Jan 17;9(1):e85422. doi: 10.1371/journal.pone.0085422 (PMC3894977; doi:10.1371/journal.pone.0085422)
Supplement: Table S3 — Genes with a fold change ≥3. (DOCX) [file pone.0085422.s007.docx]

**Table S3. (Supplementary to Figure 3) Genes with a fold change ≥ 3**

| **Group** | **Gene Symbol** | **Common and/or Official Name** |
| --- | --- | --- |
| EBV unique | CHI3L2 | YKL-39 or chitinase 3-like 2 |
|  | CD8A | CD8 |
|  | GZMH | granzyme H |
| In common with DENV | CD38 | CD38 |
|  | LMNB1 | lamin B1 |
|  | APOBEC3G | apolipoprotein B mRNA editing enzyme, catalytic polypeptide-like 3G |
|  | IDH2 | isocitrate dehydrogenase 2 (NADP+), mitochondrial |
|  | LAG3 | lymphocyte-activation gene 3 |
|  | MT1E | metallothionein 1E |
|  | E2F2 | E2F transcription factor 2 |
|  | CKS2 | CDC28 protein kinase regulatory subunit 2 |
|  | ASF1B | ASF1 anti-silencing function 1 homolog B or CIA-II |
|  | ACOT7 | BACH or acyl-CoA thioesterase 7 |
|  | MCM6 | minichromosome maintenance complex component 6 |
|  | C18orf56 | chromosome 18 open reading frame 56 |
|  | CCNB2 | cyclin B2 |
|  | CDT1 | chromatin licensing and DNA replication factor 1 |
|  | EXO1 | exonuclease 1 |
|  | AURKB | aurora kinase B |
|  | BUB1 | budding uninhibited by benzimidazoles 1 |
|  | CCNF | cyclin F or FBX1 |
|  | KIFC1 | kinesin family member C1 |
|  | TIMD4 | TIM4 or T-cell immunoglobulin and mucin domain containing 4 |
|  | CDCA7 | JPO1 or cell division cycle associated 7 |
|  | MND1 | meiotic nuclear divisions 1 homolog |
|  | SLC27A2 | solute carrier family 27 (fatty acid transporter), member 2 |
|  | GINS2 | GINS complex subunit 2 |
|  | KIAA0101 | PAF, PAF15, p15PAF, or p15(PAF) |
|  | TYMS | TS or thymidylate synthetase |
|  | STMN1 | stathmin 1 |
|  | PCNA | proliferating cell nuclear antigen |
|  | GMNN | geminin, DNA replication inhibitor |
|  | ATAD2 | ANCCA or ATPase family, AAA domain containing 2 |
|  | CENPN | centromere protein N |
|  | CDC20 | cell division cycle 20 homolog |
|  | CDCA3 | cell division cycle associated 3 |
|  | TPX2 | microtubule-associated, homolog |
|  | AURKA | aurora A or aurora kinase A |
|  | KIF14 | kinesin family member 14 |
|  | HJURP | Holliday junction recognition protein |
|  | FANCI | Fanconi anemia, complementation group I |
|  | CDCA8 | BOREALIN or cell division cycle associated 8 |
|  | BIRC5 | baculoviral IAP repeat containing 5 |
|  | CKAP2L | cytoskeleton associated protein 2-like |
|  | CDCA2 | Repo-Man or cell division cycle associated 2 |
|  | KIF2C | MCAK |
|  | POLQ | polymerase (DNA directed), theta |
|  | CDC45L | cell division cycle 45 homolog |
|  | CDCA5 | SORORIN or cell division cycle associated 5 |
|  | UBE2C | ubiquitin-conjugating enzyme E2C |
|  | MCM2 | minichromosome maintenance complex component 2 |
|  | TK1 | thymidine kinase 1, soluble |
|  | CCNA2 | cyclin A2 |
|  | ASPM | asp (abnormal spindle) homolog, microcephaly associated |
|  | APOBEC3H | apolipoprotein B mRNA editing enzyme, catalytic polypeptide-like 3H |
|  | SPC24 | NDC80 kinetochore complex component, homolog |
|  | CDC2 | CDK1 or cyclin-dependent kinase 1 |
|  | HMMR | RHAMM or hyaluronan-mediated motility receptor |
|  | MCM10 | minichromosome maintenance complex component 10 |
|  | OIP5 | LINT-25 |
|  | C12orf48 | PARPBP or PARI |
|  | WDR51A | POC1A or PIX2 |
|  | MCM4 | minichromosome maintenance complex component 4 |
|  | PLK4 | polo-like kinase 4 or SAK |
|  | NUSAP1 | nucleolar and spindle associated protein 1 |
|  | TOP2A | topoisomerase (DNA) II alpha |
|  | CENPA | centromere protein A |
|  | CHEK1 | checkpoint kinase 1 or CHK1 |
|  | STIL | SCL/TAL1 interrupting locus or SIL |
|  | CENPM | centromere protein M |
|  | CDKN3 | cyclin-dependent kinase inhibitor 3 or KAP1 |
|  | NCAPG | non-SMC condensin I complex, subunit G |
|  | PTTG3 | PTTG3P or pituitary tumor-transforming 3, pseudogene |
|  | TTK | MPS1 |
|  | FEN1 | flap structure-specific endonuclease 1 |
|  | PTTG1 | pituitary tumor-transforming 1 |
|  | MELK | maternal embryonic leucine zipper kinase |
|  | DLG7 | HURP or DLGAP5 |
|  | KIF11 | EG5 |
|  | KIF15 | HKLP2 |
|  | CEP55 | centrosomal protein 55kDa |
|  | KIF20A | MKLP2 |
|  | UBE2T | ubiquitin-conjugating enzyme E2T |
|  | POLE2 | polymerase (DNA directed), epsilon 2, accessory subunit |
|  | TRIP13 | thyroid hormone receptor interactor 13 |
|  | PSAT1 | phosphoserine aminotransferase 1 |
|  | PHGDH | phosphoglycerate dehydrogenase |
|  | PRC1 | protein regulator of cytokinesis 1 |
|  | RAD51AP1 | RAD51 associated protein 1 |
|  | RFC4 | replication factor C (activator 1) 4 |
|  | IFNG | interferon, gamma |
|  | TUBA1C | tubulin, alpha 1c |
| Common | C1QB | complement component 1, q subcomponent, B chain |
|  | IFI27 | interferon, alpha-inducible protein 27 |
|  | ANKRD22 | ankyrin repeat domain 22 |
|  | CXCL10 | IP-10 |
|  | GBP4 | guanylate binding protein 4 |
|  | GBP5 | guanylate binding protein 5 |
|  | GBP1 | guanylate binding protein 1, interferon-inducible |
|  | STAT1 | signal transducer and activator of transcription 1 |
|  | MT2A | metallothionein 2A |
|  | OASL | 2'-5'-oligoadenylate synthetase-like |
|  | PSMB9 | proteasome (prosome, macropain) subunit, beta type, 9 |
| Down in Some | IER3 | IEX1 or immediate early response gene 3 |
|  | G0S2 | G0/G1switch 2 |
|  | CXCL2 | MIP-2 |
|  | IL1B | interleukin – 1beta |
|  | IL8 | interleukin - 8 |
|  | PTGS2 | COX2 |
|  | HBEGF | heparin-binding EGF-like growth factor |
|  | OSM | oncostatin M |
|  | IRS2 | insulin receptor substrate 2 |
|  | SGK | serum/glucocorticoid regulated kinase 1 |
|  | SDPR | serum deprivation response |
