# Supplementary material for: Primary EBV Infection Induces an Expression Profile Distinct from Other Viruses but Similar to Hemophagocytic Syndromes
Source: PLoS One. 2014 Jan 17;9(1):e85422. doi: 10.1371/journal.pone.0085422 (PMC3894977; doi:10.1371/journal.pone.0085422)
Supplement: Table S4 — EBV “unique” genes. (DOCX) [file pone.0085422.s008.docx]

| **Gene Symbol** | **Common and/or Official Name** |
| --- | --- |
| ACTN1 | actinin, alpha 1 |
| ALS2CR4 | TMEM237 |
| ANG | angiogenin, ribonuclease, RNase A family, 5 |
| BATF | basic leucine zipper transcription factor, ATF-like |
| C16ORF59 | chromosome 16 open reading frame 59 |
| C17ORF53 | chromosome 17 open reading frame 53 |
| C18ORF24 | SKA1; spindle and kinetochore associated complex subunit 1 |
| C18ORF56 | chromosome 18 open reading frame 56 |
| C1ORF112 | chromosome 1 open reading frame 112 |
| C1ORF135 | AUNIP |
| C9ORF140 | SAPCD2 |
| CCNF | cyclin F; FBX1 |
| CD300LB | CD300 molecule-like family member b |
| CHAF1B | CAF1; chromatin assembly factor 1, subunit B (p60) |
| CNTNAP2 | contactin associated protein-like 2 |
| CXCL9 | chemokine (C-X-C motif) ligand 9 |
| DCLRE1A | SNM1; SNM1A; DNA cross-link repair 1A |
| DDX39 | DEAD (Asp-Glu-Ala-Asp) box polypeptide 39A |
| DEF8 | differentially expressed in FDCP 8 homolog |
| DHRS3 | dehydrogenase/reductase (SDR family) member 3 |
| DSN1 | MIND kinetochore complex component, homolog |
| ENDOGL1 | ENDOGL1; endo/exonuclease (5'-3'), endonuclease G-like |
| FLJ33590 | CXXC11 |
| GINS4 | GINS complex subunit 4 |
| GP9 | GPIX; glycoprotein IX |
| GPR162 | G protein-coupled receptor 162 |
| HMGN2 | high mobility group nucleosomal binding domain 2 |
| IL32 | interleukin 32 |
| INCENP | inner centromere protein antigens 135/155kDa |
| KCNK10 | TREK2; potassium channel, subfamily K, member 10 |
| KIF19 | kinesin family member 19 |
| LOC90925 | IGHV5-78 |
| LRP3 | low density lipoprotein receptor-related protein 3 |
| MCOLN2 | TRPML2 |
| MRPL10 | mitochondrial ribosomal protein L10 |
| NLRP12 | NALP12; NLR family, pyrin domain containing 12 |
| OBFC2B | NABP2; SSB1 |
| PAQR4 | progestin and adipoQ receptor family member IV |
| PDCD1 | PD1; programmed cell death 1 |
| PGAM1 | phosphoglycerate mutase 1 |
| PIF1 | PIF1 5'-to-3' DNA helicase homolog |
| PPP1CA | protein phosphatase 1, catalytic subunit, alpha isozyme |
| PRR11 | proline rich 11 |
| PTCRA | pre T-cell antigen receptor alpha |
| RALY | RNA binding protein, autoantigenic (hnRNP-associated with lethal yellow homolog |
| SPINT2 | HAI-2 |
| SPRED1 | sprouty-related, EVH1 domain containing 1 |
| SSBP2 | SSBP2 single-stranded DNA binding protein 2 |
| SVIL | supervillin |
| TCEAL3 | transcription elongation factor A (SII)-like 3 |
| TLR10 | toll-like receptor 10 |
| TNFRSF4 | OX40 |
| TSPAN18 | tetraspanin 18 |
| TSPAN9 | tetraspanin 9 |
| TXNL2 | PICOT; GRX3; glutaredoxin 3 |
| USP54 | ubiquitin specific peptidase 54 |
| VENTX | VENT homeobox |
| VWF | von Willebrand factor |
| ZDHHC1 | zinc finger, DHHC-type containing 1 |

**Table S4.** Lists genes changed during EBV infection with a fold change ≥ 2 and a *p* value of ≤ 0.05, but also had an average fold change < 1.5 in all other viral infections. The gene symbol and the common or official name for the gene are shown.
